# Single molecule aggregation-induced dual and white-light emissive etherified aroyl-*S*,*N*-ketene acetals *via* one-pot synthesis†

**DOI:** 10.1039/d3ra02935b

**Published:** 2023-06-05

**Authors:** Lukas Biesen, Thomas J. J. Müller

**Affiliations:** a Institut für Organische Chemie und Makromolekulare Chemie, Heinrich-Heine-Universität Düsseldorf Universitätsstraße 1 D-40225 Düsseldorf Germany ThomasJJ.Mueller@hhu.de

## Abstract

Etherified aroyl-*S*,*N*-ketene acetals are readily synthesized by a novel one-pot addition–elimination-Williamson-etherification sequence. Although the underlying chromophore remains constant, derivatives show pronounced color-tuning of solid-state emission and AIE characteristics, whereas a hydroxy-methyl derivative represents an easily accessible mono molecular aggregation-induced white-light emitter.

## Abstract 2

A novel addition–elimination-Williamson-etherification one-pot sequence furnishes rainbow-tunable solid-state emissive etherified aroyl-*S*,*N*-ketene acetals that show aggregation-induced white light emission (AIWLE).

## Graphical abstract

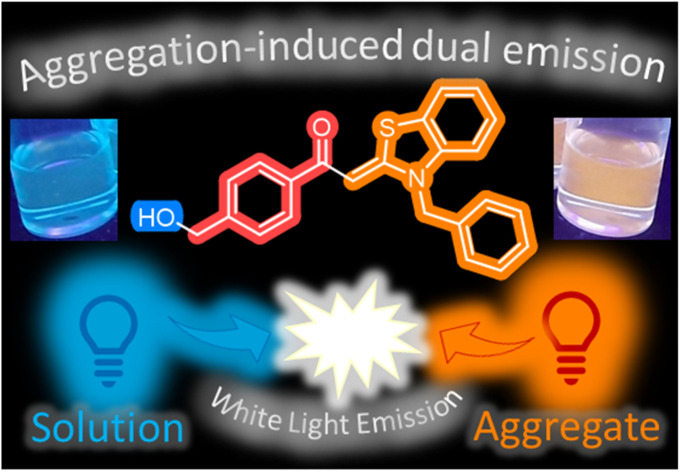

The generation of white-light emission remains to this day the holy grail for many materials chemists.^1^ Classically, white light emission in devices is generated *via* a mixture of blue, green and red emitters in the respective active layer of the device.^1*a*,2^ With growing interest in white light emission for various applications like OLEDs,^3^ in thin films and displays^4^ or for molecular sensors and switches,^5^ the desire to achieve white light emission from a single emitter molecule steadily increases.^6^

While the goal is clear the ways to get white light emission according to CIE (Commission internationale de l'éclairage) 1931 color space are multifaceted. Many concepts have been applied like harvesting self-assembly processes,^5*a*,7^ exciplex formation,^8^ phosphorescent materials,^9^ and environmental influences like solvent effects^10^ or protonation.^11^ But one of the most often used concepts for an efficient white light emission is the development of bi- or multichromophoric systems in which different emitter molecules are covalently linked but not conjugated. These materials can emit individually in the absence of energy transfer or as a consequence of a frustrated, *i.e.* partial energy transfer.^12^ For a dual (or multiple) emission pathway to occur, originating from the same molecule, it is mandatory that a partial suppression of excited state resonance energy transfer processes between the constituting luminophores takes place.^13^ Prime methodologies for white light emission causing dual emission characteristics are excited state intramolecular proton transfer processes (ESIPT)^14^ or, especially in recent years, aggregation-induced emission (AIE) effects^15^ which rely on the restriction of intramolecular motions and the deactivation of nonradiative pathways.^16^ For dual emitting systems, the subsequent term aggregation-induced dual emission (AIDE) has been coined.^17^ However, the synthesis of bi- or multichromophoric systems is often complex^18^ and, as we have seen in previous works with bichromophoric emitters, it is not guaranteed that the dual emission is perfectly tuned for a white light emission to occur.^19^ Therefore, single molecule white light emitters based on AIE properties rank high as relevant target structures.^1*e*,6*a*,20^ Consequently, we returned from bi- and multichromophoric aroyl-*S*,*N*-ketene acetal systems to the core structural motif for a new approach to white light emitters. Key feature of aroyl-*S*,*N*-ketene acetals are their versatility, their easy accessibility and their comprehensive tunability, thus promoting them as prime examples for the next generation of white light emitters.

During synthesis of chloromethyl substituted aroyl-*S*,*N*-ketene acetal 3, we observed a subsequent Williamson etherification with the co-solvent ethanol to give ethyl ether 4g.^21^ Therefore, we became interested in exploiting this concept and established a novel one-pot condensation-etherification process by applying the standard protocol for the generation of aroyl-*S*,*N*-ketene acetals^22^ and varying the alcohol. The alcohol (ROH) has been used in a great excess as it simultaneously acts as reaction partner and as a co-solvent. The surplus of triethylamine ensures that it can act as a base for both, the addition elimination, and the Williamson etherification step. This method leads to the formation of 10 etherified aroyl-*S*,*N*-ketene acetals 4 in yields ranging between 25 and 92% after a single flash chromatography purification step (for details, see ESI†). It is possible to use benzyl alcohol as well as various aliphatic alcohols, alkynols, alkenols and phenols (Scheme 1).

**Scheme 1:**
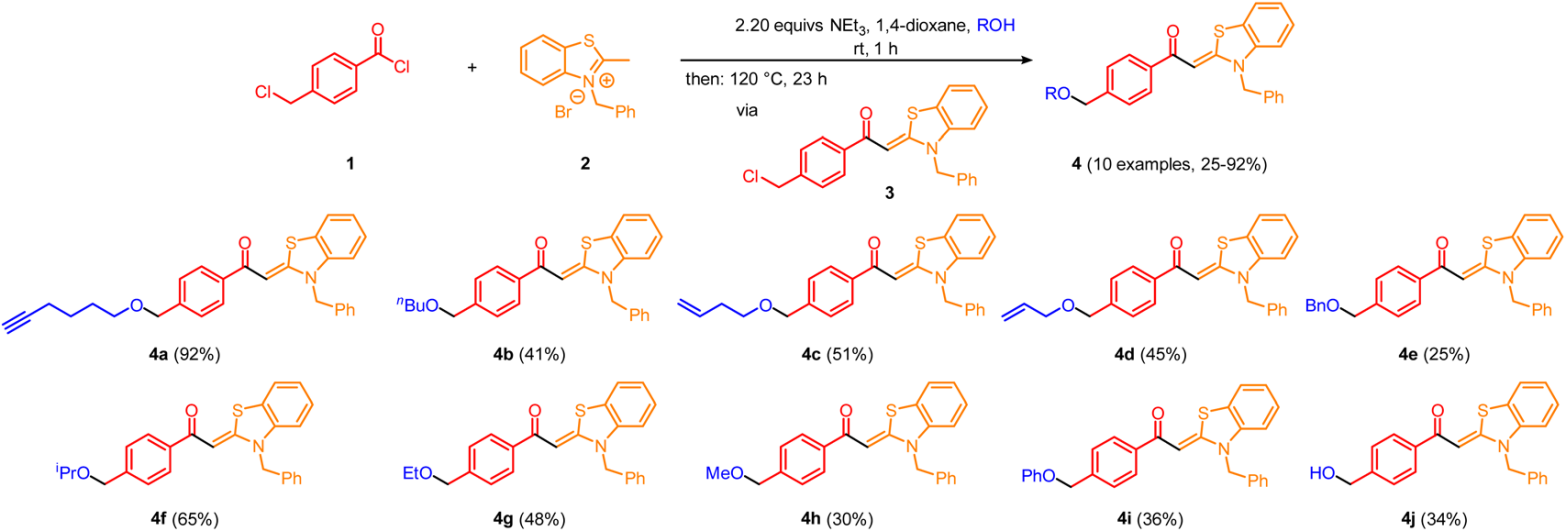
Synthesis of etherified aroyl-*S*,*N*-ketene acetals 4. (All reactions were performed on 1.0 mmol scale: 1 (1.0 mmol), 2 (1.1 mmol), triethylamine (2.2 mmol) in 1,4-dioxanes/alcohol 5 : 2 (7.0 mL) were stirred at room temp for 1 h and at 120 °C for 23 h. Yields after flash chromatography on silica gel.)

For the implementation of water into the one-pot sequence, potassium hydroxide was added as an additional base and a hydroxide ion source in the synthesis of derivative 4j.

Under day light, all compounds are yellow to red solids, or in some cases resins and oils. Characteristic absorption maxima *λ*_abs_ for all derivatives range in typical margins for aroyl-*S*,*N*-ketene acetals between 385 and 387 nm and respective absorption coefficients add up to 41 300 L mol^−1^ cm^−1^ at maximum. Etherified aroyl-*S*,*N*-ketene acetals 4 only luminesce very weakly blue (*λ*_em_ = 447 nm) in solution but show easily distinguishable solid-state emission, yet, again with tunable emission color ranging from green (4f, *λ*_em_ = 500 nm) over yellow (4c, *λ*_em_ = 547 nm) to red (4j, *λ*_em_ = 618 nm) thus encompassing a region of nearly 4000 cm^−1^ (Fig. 1). The solid-state emission was only reported for solid products (4c, 4d, 4f, 4g, and 4j), and oils or resins (4a, 4b, 4e, 4h, and 4i) did not fluoresce in these states.

**Fig. 1:**
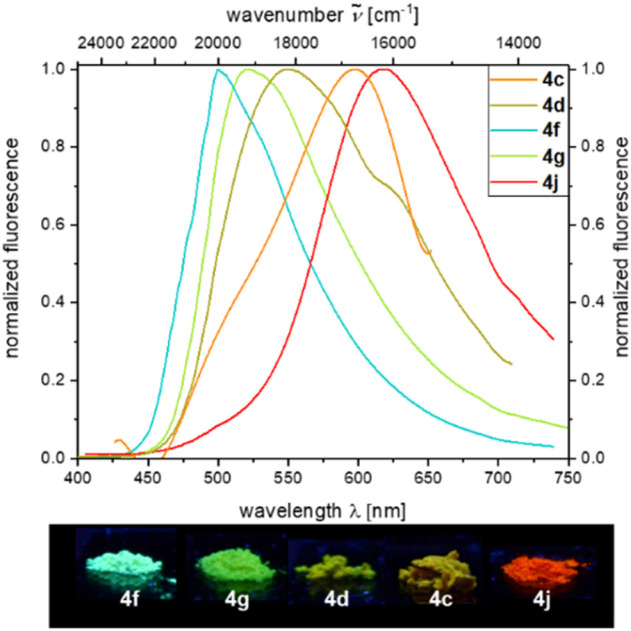
Top: Normalized solid-state emission spectra of etherified aroyl-*S*,*N*-ketene acetals 4 (*λ*_exc_ = *λ*_abs,max_ at *T* = 298 K); bottom: solid-state fluorescence colors of selected aroyl-*S*,*N*-ketene acetal derivatives 4 (*λ*_exc_ = 365 nm) revealing a rainbow tuning of solid state emission color.

This is quite remarkable as the ether substituent is not conjugated and therefore does not directly affect the chromophoric system. Nevertheless, the individual residues differ from each other to a significant extent. In previous works,^21^ we could unequivocally show that the intermolecular distance of the chromophores in the solid also has an significant influence on the solid-state emission properties, which also applies to the etherified aroyl-*S*,*N*-ketene acetals 4. Quantum yields are comparably low, and the isopropoxy-substituted derivative 4f reveals the highest *Φ*_f_ with 0.07.

Etherified aroyl-*S*,*N*-ketene acetals 4 all show aggregation-induced enhanced emission (AIEE) encompassing a significant bathochromic shift of emission maximum upon aggregation. It spans from weak blue luminescence in ethanol to yellow to orange luminescence in the aggregated state which reaches its maximum at water fractions of 90% (for details, see ESI†). The emission intensity is increased four-to sixfold upon aggregation. For many derivatives, *e.g.* butoxy substituted compound 4b or benzyloxy substituted derivative 4e, distinct dual emission with two pronounced emission maxima at around 520 and 610 nm is discernable. This dual emission may be ascribed to the simultaneous occurrence of different aggregate forms,^17*b*,23^ since only a single chromophore system of the aroyl-*S*,*N*-ketene acetal is present. Different aggregate shapes and morphologies as well as aggregate sizes can cause different emission behavior. In case of compound 4b, according to this hypothesis, both aggregates are simultaneously present throughout the induced aggregation; in the case of compound 4e, both aggregate forms are initially simultaneously present until one aggregate form finally dominates at higher water contents (Fig. 2). Additionally, aspects like hydrophobicity of the substituents influence the emission intensity and starting point of aggregation.

**Fig. 2:**
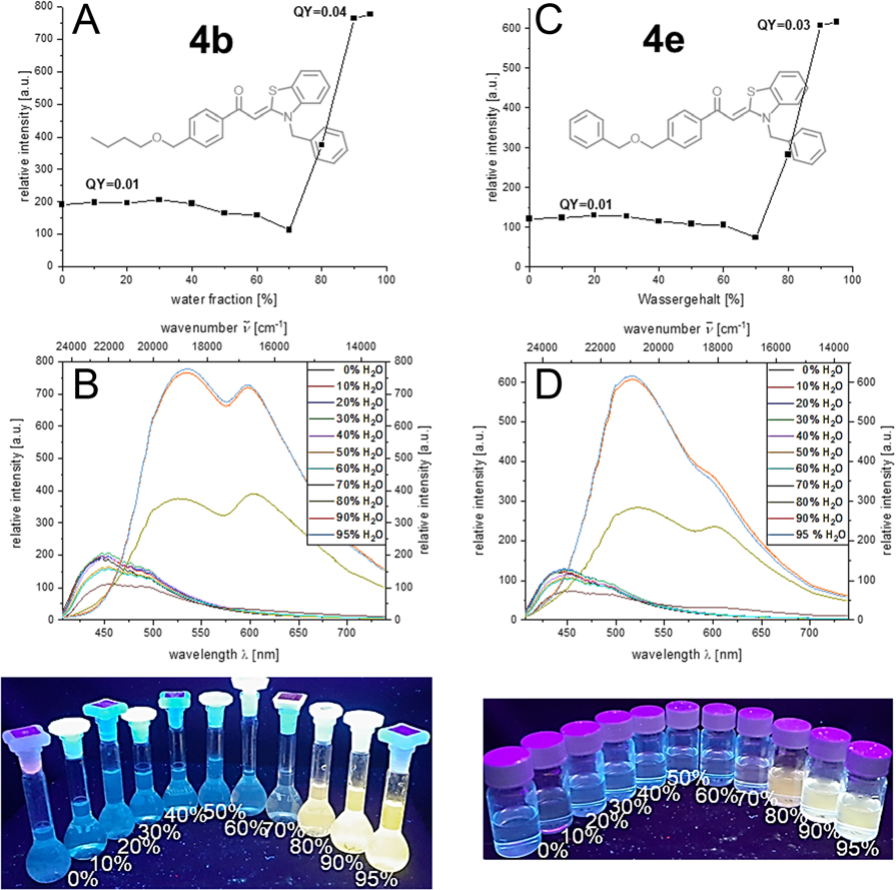
Top: Emission intensity of 4b (A) and 4e (C); 2nd row: emission spectra of 4b (B) and 4e (D) in ethanol/water mixtures upon increasing water content (recorded at *T* = 298 K, *c* = 10^−7^ M, *λ*_exc_ = *λ*_abs,max_); bottom: visualization of 4b (left) and 4e (right) in ethanol/water mixtures with increasing water content (*λ*_exc_ = 365 nm).

Hydroxymethyl derivative 4j follows the same general trend. At the beginning of the induced aggregation, at low water contents, the solution luminesces blue (*λ*_em_ = 454 nm, *Φ*_f_ = 0.02), and the emission intensity decreases due to the polarity increase up to a water content of 60%. With further increase of the water content, the emission intensity increases and, after a plateau between 80 and 90% water content the maximum is reached at 95%, and a fluorescence quantum yield of *Φ*_f_ 0.03 can be detected. Simultaneously, with the appearance of the aggregates, a second emission maximum grows in at *λ*_em_ = 615 nm. With beginning aggregation, an intensity alignment of the two emission bands can be seen. With progressing aggregation, the intensity of the emission band at *λ*_em_ = 454 nm decreases until it completely disappears at 95% water content, while the intensity of the emission band at *λ*_em_ = 615 nm steadily increases. At water fractions between 60 and 70%, both emission bands clearly coexist and show equal intensity. The change from blue to red fluorescence suggests white light emission by color mixing in the course of aggregation. Therefore, water/ethanol mixtures with variation in 2% water steps were prepared and analyzed by CIE analysis to determine the corresponding tristimulus coordinates of the emission.

Indeed, the white light point is traversed in the course of induced aggregation. At water contents of 65 and 66% white light emission can be unequivocally assigned upon trespassing the white point. Thus, 4-(hydroxymethyl)-aroyl-*S*,*N*-ketene acetal 4j represents the first aggregation-induced monomolecular white-light emitter of this class of compounds (Fig. 3).

**Fig. 3:**
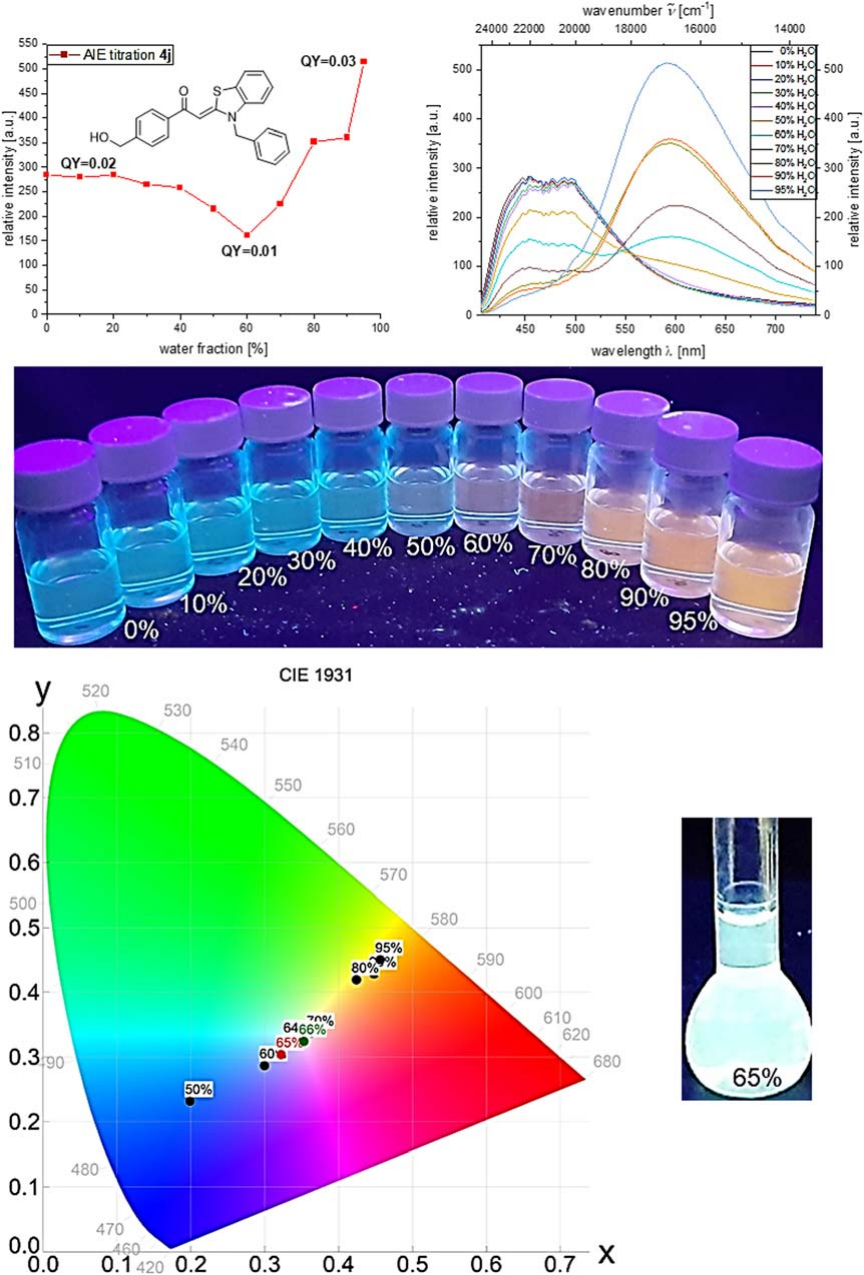
Top: Emission intensity (left) and emission spectra (right) of 4j in ethanol/water mixtures upon increasing water content (recorded at *T* = 298 K, *c* = 10^−7^ M, *λ*_exc_ = *λ*_abs,max_); 2nd row: visualization of 4j in ethanol/water mixtures with increasing water content (*λ*_exc_ = 365 nm); bottom: CIE diagram of emission maxima of 4j in ethanol/water mixtures and white light emission at a water fraction of 65% (increased concentration *c* = 10^−3^ M).

The deviating behavior of compound 4j is caused by the hydroxymethyl substituent. The formation of intermolecular hydrogen bonds appears to be a plausible rationale. Although the carbonyl group of aroyl-*S*,*N*-ketene acetals can act as a hydrogen bond acceptor in all derivatives, the hydroxymethyl substituted aroyl-*S*,*N*-ketene acetal 4j can additionally serve as a potent hydrogen bond donor due to the hydroxymethyl group. This behavior is observed to a lesser extent for phenolic aroyl-*S*,*N*-ketene acetals in previous studies for which we observed a strictly derivative AIE behavior compared to other aroyl-*S*,*N*-ketene acetal derivatives.^21^

While at lower water fractions the interactions and hydrogen bonding between the chromophore 4j and the solvent particularly dominate, with increasing aggregation of the dye molecules, the intermolecular hydrogen bonds between the individual molecules grow much more significantly as exemplarily shown in Scheme 2.

**Scheme 2:**
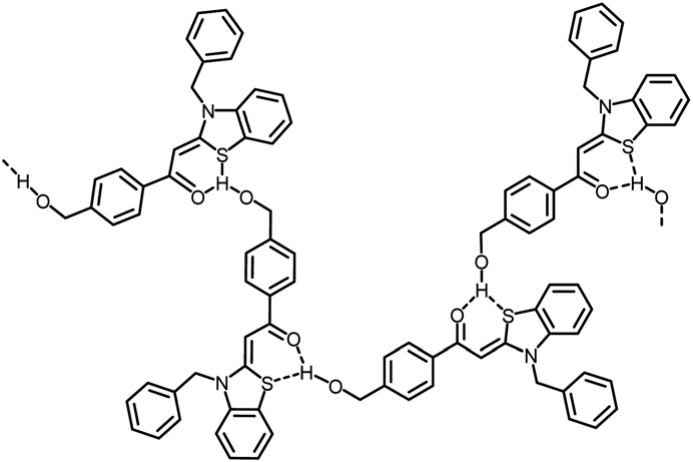
Proposed example for intermolecular hydrogen bonding of molecules 4j upon aggregation.

These interactions lead to a bathochromic shift of the emission and cause a dominance of red fluorescence at high water contents. A similar effect has already been mentioned frequently in the literature.^24^

In summary, a novel one-pot synthesis furnishes the class of etherified aroyl-*S*,*N*-ketene acetals in moderate to excellent yields. The absorption spectra match nearly perfectly with the spectra of aroyl-*S*,*N*-ketene acetal core motifs and although the ether substituents are not conjugated, they nevertheless allow for a comprehensive tuning of solid state emission color from green to red. Aggregation studies revealed a pronounced AIEE behavior for all compounds with a color shift from blue to yellowish-orange with an accompanying dual emission. For hydroxymethyl-substituted compound 4j, this aggregation-induced dual emission led to white light emission very likely caused by intermolecular hydrogen bonds in juxtaposition with the AIEE properties. This allows for the generation of an easily accessible, monomolecular white light emitting aroyl-*S*,*N*-ketene acetal. Based on this rationale and the respective emission properties, works regarding this type of white light emitter for increasing the quantum yield are currently underway.

## Conflicts of interest

There are no conflicts to declare.

## Supplementary Material

RA-013-D3RA02935B-s001
