# Supplementary material for: Single molecule aggregation-induced dual and white-light emissive etherified aroyl-S,N-ketene acetals via one-pot synthesis
Source: RSC Adv. 2023 Jun 5;13(25):16867–71. doi: 10.1039/d3ra02935b (PMC10240259; doi:10.1039/d3ra02935b)
Supplement: RA-013-D3RA02935B-s001 [file RA-013-D3RA02935B-s001.pdf]

Electronic Supplementary Material (ESI) for RSC Advances.
This journal is © The Royal Society of Chemistry 2023

Single molecule aggregation-induced dual and white-light emissive etherified aroyl-*S,N*-ketene acetals via one-pot synthesis

Lukas Biesen and Thomas J. J. Müller*

Institut für Organische Chemie und Makromolekulare Chemie, Heinrich-Heine-Universität Düsseldorf, Universitätsstraße 1, D-40225 Düsseldorf, Germany.

E-Mail: ThomasJJ.Mueller@uni-duesseldorf.de

Fax: (+)49 (0)211-8114324

Table of contents

1 General considerations 2

2 Overview of synthesized etherified aroyl-S,N-ketene acetals 4 4

3 Starting material 6

3-Benzyl-2-methylbenzo[*d*]thiazol-3-iumbromide (**2**)[3] 6

(*Z*)-2-(3-Benzyl)benzo[*d*]thiazol-2(3*H*)-ylidene)-1-(4-chloromethylphenyl)ethan-1-one (**3**)[4],[5] .. 7

3.1 Synthesis and analytical data of etherified aroyl-*S,N*-ketene acetals 4 8

3.1.1 General procedure I (**GPI**) for the synthesis of etherified aroyl-*S,N*-ketene acetals **4** 8

3.1.1.1 Spectroscopic data 11

(*Z*)-2-(3-Benzylbenzo[*d*]thiazol-2(3*H*)-ylidene)-1-(4-((hex-5-yn-1-yloxy)methyl)phenyl)ethan-1-one (**4a**) 11

(*Z*)-2-(3-Benzylbenzo[*d*]thiazol-2(3*H*)-ylidene)-1-(4-(butoxymethyl)phenyl)ethan-1-one (**4b**) . 12

(*Z*)-2-(3-Benzylbenzo[*d*]thiazol-2(3*H*)-ylidene)-1-(4-((but-3-en-1-yloxy)methyl)phenyl)ethan-1-one (**4c**) 13

(*Z*)-1-(4-((Allyloxy)methyl)phenyl)-2-(3-benzylbenzo[*d*]thiazol-2(3*H*)-ylidene)ethan-1-one (**4d**) 14

(*Z*)-2-(3-Benzylbenzo[*d*]thiazol-2(3*H*)-ylidene)-1-(4-((benzyloxy)methyl)phenyl)ethan-1-one (**4e**) 15

(*Z*)-2-(3-Benzylbenzo[*d*]thiazol-2(3*H*)-ylidene)-1-(4-(isopropoxymethyl)phenyl)ethan-1-one (**4f**) 16

(*Z*)-2-(3-Benzylbenzo[*d*]thiazol-2(3*H*)-ylidene)-1-(4-(ethoxymethyl)phenyl)ethan-1-one (**4g**) . 17

(*Z*)-2-(3-Benzylbenzo[*d*]thiazol-2(3*H*)-ylidene)-1-(4-(methoxymethyl)phenyl)ethan-1-one (**4h**) 18

(*Z*)-2-(3-Benzylbenzo[*d*]thiazol-2(3*H*)-ylidene)-1-(4-(phenoxymethyl)phenyl)ethan-1-one (**4i**) 19

(*Z*)-2-(3-Benzylbenzo[*d*]thiazol-2(3*H*)-ylidene)-1-(4-(hydroxymethyl)phenyl)ethan-1-one (**4j**) 20

3.2 NMR spectra of etherified aroyl-*S,N*-ketene acetals 4 21

4 Overview of photophysical properties of etherified aroyl-S,N-ketene acetals 4 31

5 Absorption and emission spectra 33

5.1 Absorption and emission spectra of etherified aroyl-*S,N*-ketene acetals 4 33

6 Literature 46

1 General considerations

Reactions were carried out in dried and sintered Schlenk tubes or round bottom flasks under nitrogen atmosphere. Solvents were dried by a solvent purification system *MB-SPS-800* of the company *MBraun Inertgas-Systeme GmbH*.
The used chemicals which have not been synthesized were purchased at *Acros Organics BVBA, Alfa Aeser GmbH & Co KG, Fluorochem Ltd., J&K Scientific Ltd., Merck KGaA, Macherey-Nagel GmbH & Co. KG, Sigma-Aldrich Chemie GmbH* and *VWR* and have been used without further purification. The solvents ethanol and tetrahydrofuran (THF) (spectroscopic grade) were purchased from *Merck* and *Sigma-Aldrich* (Germany), respectively. Milli-Q-water was obtained from a *Millipore water purification system*. Carboxylated 8 µm-sized polystyrene particles (PSP) were obtained from *Kisker Biotech GmbH* (Germany).

Further purification of the compounds was performed by flash column chromatography (silica gel M60 pore size 0.040-0.063 nm) of the company *Macherey-Nagel*. The crude product was adsorbed on Celite®545 of the company *Carl Roth GmbH*, placed on the suspended silica gel and purified with a positive pressure of 2 bar. Distilled solvent mixtures of *n*-hexane, acetone and methanol have been used as eluents.

The control of reaction progress was done via thin layer chromatography (TLC) with silica coated aluminium plates F_{254}, of the company *Macherey-Nagel GmbH & Co. KG.*

The melting points have been measured with *Melting Point B-540* of the company *Büchi* according to the protocol of *Kofler*.[1]

^{1}H,^{13}C and DEPT 135-spectra have been measured at 298 K on an *Avance III - 300* and an *Avance III - 600* of the company *Bruker*. Chemical shifts in the ^{1}H and ^{13}C NMR are reported in ppm relative to deuterated solvents such as acetone-d$_6$ (δ_H 2.05, δ_C 29.84, δ_C 206.26) with CS_2 (δ_C 192.28) and DMSO-d$_6$ (δ_H 2.50, δ_C 39.51).[2] The multiplicity is abbreviated as followed: s = singulet; d = doublet; t = triplet; td = triplet of doublet; dd = doublet of doublet; dt = doublet of triplet, dq = doublet of quartet; pd = quintet of doublet m = multiplet. The assignment of primary carbon centers (CH), secondary carbon centers (CH_2), tertiary carbon centers (CH_3) and quaternary carbon centers (C_{quat}) were made by using DEPT-135 spectra.
All mass spectrometry experiments have been performed by the department for mass spectrometry of the University of Düsseldorf (HHUCeMSA). EI mass spectra have been measured with Triple-Quadrupol-spectrometer *TSQ 7000* of the company *Finnigan MAT*. MALDI spectra have been measured with a *MALDI/TOF UltrafleXtreme* of the company *Bruker Daltronik*.

IR spectra were recorded with neat compounds under attenuated total reflection (ATR) with *IRAffinity-1* of the company *Shimadzu* and the intensities were characterized as strong (s), middle (m) and weak (w).

The elementary analyses have been measured with *Perkin Elmer Series II Analyser 2400* or *Vario Micro Cube* of the company *Analysensysteme GmbH* at the microanalytical laboratory of the institute for Pharmaceutical and Medicinal Chemistry of the University Düsseldorf.

UV/Vis spectra of the dye solutions were measured with a *Lambda 19* spectrometer from *Perkin Elmer*. The emission spectra of the dye solutions and the solid compounds were recorded with a *Hitachi F-7000* spectrofluorometer using the emission correction curve provided by the instrument manufacturer. Emission spectra were not corrected for the wavelength-dependent spectral responsivity of the fluorometer. All solution spectra were recorded with dyes dissolved in spectroscopic grade solvents at 298 K using 1 cm-$_{quat}$z cuvettes from *Hellma GmbH*. The molar extinction coefficients of dye solutions of known dye concentration were determined by five-point regression line.

2 Overview of synthesized etherified aroyl-*S,N*-ketene acetals 4

Table S1: Synthesis of etherified aroyl-*S,N*-ketene acetals **4**.

Entry	ROH	Yield of product **4** (%)
1	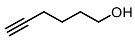	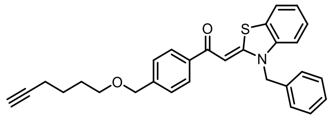 **4a** (92)
2	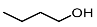	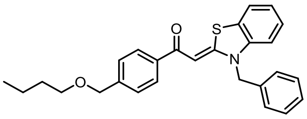 **4b** (41)
3	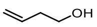	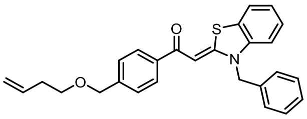 **4c** (51)
4	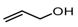	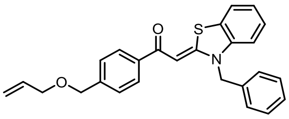 **4d** (45)
5	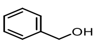	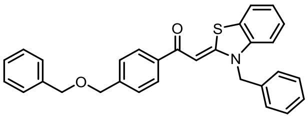 **4e** (25)
6	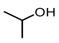	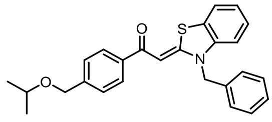 **4f** (65)
7	EtOH	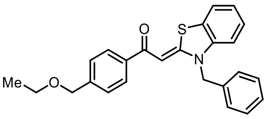 **4g** (48)

Table S1 continued.

Entry	ROH	Yield of product **4** (%)
8	MeOH	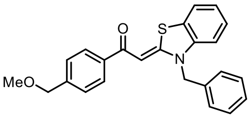 **4h** (30)
9	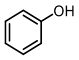	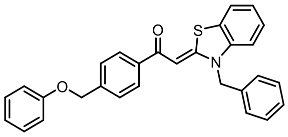 **4i** (36)
10	H_2O	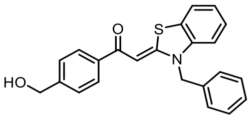 **4j** (34)

3 Starting material

3-Benzyl-2-methylbenzo[*d*]thiazol-3-iumbromide (2)[3]

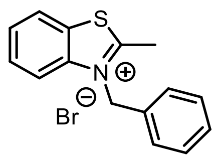

$C_{15}H_{14}BrNS$

[319.00]

2-Methylbenzothiazole (7.45 g, 50.0 mmol) and benzylbromide (10.2 g, 60.0 mmol) were placed in a round-bottom flask. The reaction mixture was stirred at 75 °C for 20 h, until the solution was completely hardened. The formed solid was filtrated via a Buechner funnel, washed with diethyl ether and dried under vacuo. The synthesis yielded 12.4 g (39.0 mmol, 78%) of the desired product **2** as a pink solid.

Mp: 200 °C.

R_f (*n*-hexane/acetone 4:1): 0.10.

^{1}H NMR (300 MHz, DMSO-d$_6$): δ 3.30 (s, 3 H), 6.14 (s, 2 H), 7.33-7.40 (m, 5 H), 7.80 (pd, ^3J = 7.5 Hz, ^4J = 1.9 Hz, 2 H), 8.23 (dd, ^3J = 7.8 Hz, ^4J = 1.4 Hz, 1 H), 8.56 (dd, ^3J = 7.4 Hz, ^4J = 1.8 Hz, 1 H).

^{13}C NMR (75 MHz, DMSO-d$_6$): δ 17.5 (CH_3), 51.9 (CH_2), 117.1 (CH), 127.0 (CH), 128.3 (CH), 128.5 (CH), 129.1 (CH), 129.2 (C_{quat}), 129.5 (CH), 132.8 (C_{quat}), 140.8 (C_{quat}), 178.3 (C_{quat}).

EI + MS (70 eV, *m/z* (%)): 240 (15), 239 ($[C_{15}H_{14}NS]^+$, 67), 238 (65), 224 ($[C_{14}H_{10}NS]^+$, 14), 162 ($[C_9H_8NS]^+$, 13), 148 ($[C_8H_6NS]^+$, 32), 104 ($[C_7H_6N]^+$, 12), 91 ($[C_7H_7]^+$, 100), 65 ($[C_5H_5]^+$, 23).

(*Z*)-2-(3-Benzyl)benzo[*d*]thiazol-2(3*H*)-ylidene)-1-(4-chloromethylphenyl)ethan-1-one (3)[4],[5]

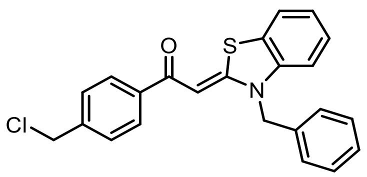

$C_{23}H_{18}ClNOS$

[391.08]

4-(Chloromethyl)benzoyl chloride **1** (0.189 g, 1.00 mmol) and benzothiazolium salt **2** (0.351 g, 1.10 mmol) were placed in a sintered, dry screw-cap Schlenk-tube under nitrogen atmosphere and dissolved in 5 mL dry 1,4-dioxane. Triethylamine (0.300 mL, 2.20 mmol) was added to the reaction mixture and the solution was stirred for 1 h at room temperature. Thereafter, the reaction mixture was stirred at 120 °C (oil bath) for 23 h. The crude product was absorbed onto Celite® and purified by flash chromatography on silica gel (*n*-hexane/acetone 3:1). The product was suspended in *n*-hexane, the sediment was filtrated and dried under vacuo. The synthesis yielded 0.230 g (0.588 mmol, 59%) of the desired product **3** as a yellow solid.

Mp: 186 °C.

R_f (*n*-hexane/acetone 3:1): 0.47.

^{1}H NMR (300 MHz, acetone-d$_6$): δ 4.71 (s, 2 H), 5.59 (s, 2 H), 6.87 (s, 1 H), 7.21-7.38 (m, 8 H), 7.45-7.50 (m, 2 H), 7.76 (d, ^3J = 7.8 Hz, 1 H), 7.91-7.96 (m, 2 H).

^{13}C NMR (75 MHz, acetone-d$_6$): δ 46.4 (CH_2), 49.7 (CH_2), 88.3 (CH), 111.5 (CH), 123.2 (CH), 123.8 (CH), 127.5 (CH), 128.0 (C_{quat}), 128.3 (CH), 128.4 (CH), 128.5 (CH), 129.7 (CH), 136.1 (C_{quat}), 140.4 (CH), 140.9 (C_{quat}), 141.1 (C_{quat}), 162.6 (C_{quat}), 183.6 (C_{quat}).

MALDI-TOF (*m/z*): 394.3 ($C_{23}H_{18}{}^{37}ClNOS+H^+$), 392.3 ($C_{23}H_{18}{}^{35}ClNOS+H^+$).

IR $\tilde{\nu}$ [cm^{-1}]: 646 (m), 665 (m), 689 (m), 718 (s), 739 (m), 743 (s), 772 (m), 793 (m), 826 (m), 878 (m), 908 (m), 939 (w), 978 (w), 1016 (m), 1042 (m), 1065 (m), 1090 (m), 1134 (w), 1157 (w), 1177 8m), 1194 (m), 1229 (m), 1263 (m), 1290 (w), 1304 (m), 1331 (m), 1339 (m), 1356 (w), 1414 (w), 1454 (s), 1485 (s), 1510 (m), 1558 (m), 1599 (m), 2336 (w), 2868 (w), 3028 (w), 3061 (w).

UV/Vis (C_3H_6O): λ_{max} (ε) = 387 (42100).

Anal calcd for $C_{23}H_{18}ClNOS$ [391.1]: C 70.49, H 4.63, N 3.57, S 8.18; Found: C 70.46, H 4.71, N 3.46, S 8.13.

3.1 Synthesis and analytical data of etherified aroyl-*S,N*-ketene acetals 4

3.1.1 General procedure I (GPI) for the synthesis of etherified aroyl-*S,N*-ketene acetals 4

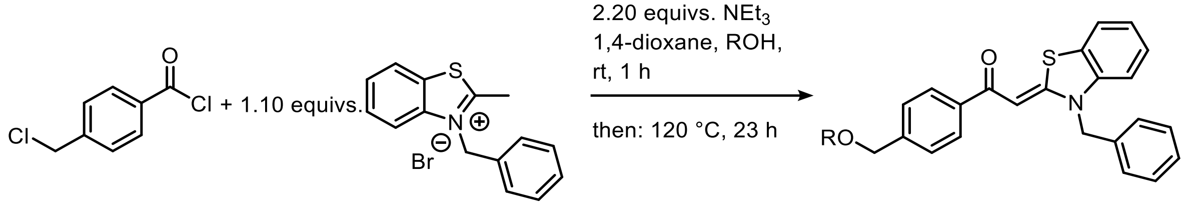

1 **2** **4**

4-(Chloromethyl)benzoyl chloride **1** (0.189 g, 1.00 mmol) and benzothiazolium salt **2** (0.351 g, 1.10 mmol) were placed in a sintered, dry screw-cap Schlenk-tube under nitrogen atmosphere and dissolved in 5 mL dry 1,4-dioxane and 2 mL alcohol. Triethylamine (0.300 mL, 2.20 mmol) was added to the reaction mixture and the solution was stirred for 1 h at room temperature. Thereafter, the reaction mixture was stirred at 120 °C (oil bath) for 23 h. The crude product was absorbed onto Celite® and purified by flash chromatography on silica gel (*n*-hexane/acetone). The product was suspended in *n*-hexane, the sediment was filtrated and dried under vacuo.

Table S2: Experimental details for the synthesis of etherified aroyl-*S,N*-ketene acetals **4**.

Entry	ROH	Yield of product **4** [g] (%)
1	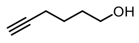	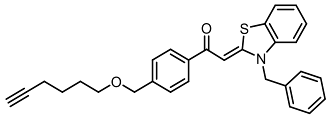<br>0.415 (92) of **4a**
2	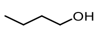	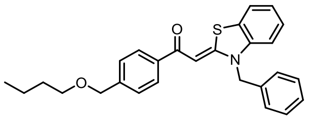<br>0.178 (41) of **4b**
3	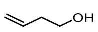	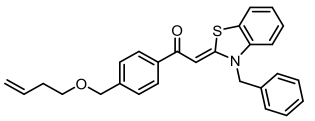<br>0.217 (51) of **4c**
4	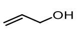	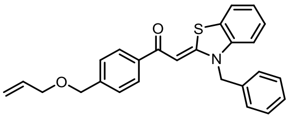<br>0.178 (45) of **4d**
5	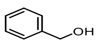	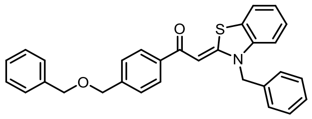<br>0.116 (25) of **4e**
6	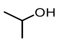	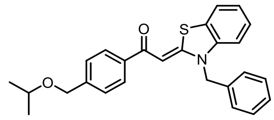<br>0.262 (65) of **4f**
7	EtOH	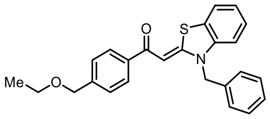<br>0.193 (48) of **4g**
8	MeOH	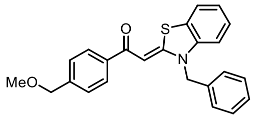<br>0.115 (30) of **4h**

Table S2 continued.

Entry	ROH	Yield of product **4** [g] (%)
9	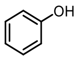	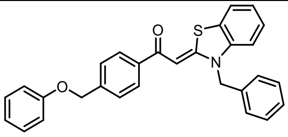 0.163 (36) of **4i**
10[(a)]	H_2O	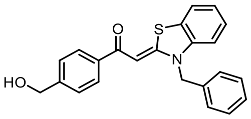 0.128 (34) of **4j**

(a) For this reaction, 1.00 g of potassium hydroxide was added to the reaction mixture.

3.1.1.1 Spectroscopic data

(*Z*)-2-(3-Benzylbenzo[*d*]thiazol-2(3*H*)-ylidene)-1-(4-((hex-5-yn-1-yloxy)methyl)phenyl)ethan-1-one (4a)

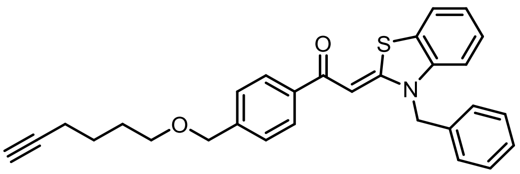

$C_{29}H_{27}NO_2S$

[453.18]

The synthesis was performed according to **GPIII** using *n*-hexane/acetone 3:1 as eluent for column chromatography to give 0.415 g (0.916 mmol, 92%) of **4a** as a yellow resin.

R_f (*n*-hexane/acetone 3:1): 0.40.

^{1}H NMR (300 MHz, acetone-d_6): δ 1.51-1.66 (m, 4 H), 2.16-2.22 (m, 2 H), 2.30 (t, ^3J = 2.8 Hz, 2 H), 3.55 (t, ^3J = 6.2 Hz, 4 H), 5.63 (s, 2 H), 6.94 (s, 1 H), 7.23-7.45 (m, 7 H), 7.48-7.52 (m, 1 H), 7.69-7.85 (m, 1 H), 7.90-8.08 (m, 3 H).

^{13}C NMR (75 MHz, acetone-d_6): δ 18.6 (CH_2), 25.9 (CH_2), 32.7 (CH_2), 49.7 (CH_2), 61.8 (CH_2), 69.8 (CH_2), 85.0 (CH_2), 95.5 (CH), 111.5 (CH), 123.2 (CH), 123.3 (CH), 123.8 (CH), 127.5 (CH), 127.9 (CH), 128.3 (CH), 128.6 (CH), 128.9 (C_{quat}), 129.5 (CH), 129.8 (CH), 131.4 (CH), 136.3 (C_{quat}), 141.0 (C_{quat}), 154.2 (C_{quat}), 154.6 (C_{quat}), 164.5 (C_{quat}), 185.6 (C_{quat}).

MALDI-TOF (*m/z*): 454.2 ($C_{29}H_{27}NO_2S+H^+$).

IR $\tilde{\nu}$ [cm^{-1}]: 629 (s), 700 (m), 733 (m), 818 (w), 843 (w), 860 (w), 878 (w), , 924 (m), 937 (m), 964 (m), 989 (m), 1040 (m), 1061 (m), 1121 (w), 1177 (m), 1203 (m), 1271 (m), 1312 (m), 1329 (m), 1354 (w), 1391 (w9k, 1412 (m), 1433 (m), 1454 (m), 1557 (w), 1589 (w), 1607 (w), 1651 (w), 1665 (m), 1721 (m), 2116 (w), 2866 (w), 2940 (w), 3034 (w), 3065 (w), 3154 (w), 3296 (w).

UV/Vis (C_3H_6O): λ_{max} (ε) = 385 (6700).

HRMS (ESI) calcd. for $C_{29}H_{27}NO_2S+H^+$: 453.1762; Found: 462.1760.

(*Z*)-2-(3-Benzylbenzo[*d*]thiazol-2(3*H*)-ylidene)-1-(4-(butoxymethyl)phenyl)ethan-1-one (4b)

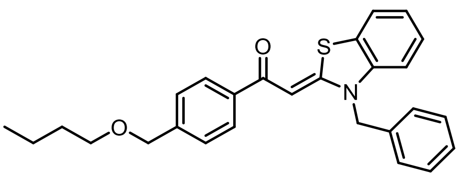

$C_{27}H_{27}NO_2S$

[429.17]

The synthesis was performed according to **GPIII** using *n*-hexane/acetone 3:1 to 1:1 as eluent for column chromatography to give 0.178 g (0.414 mmol, 41%) of **4b** as a yellow resin.

R_f (*n*-hexane/acetone 3:1): 0.46.

^{1}H NMR (300 MHz, acetone-d$_6$): δ 0.90 (td, ^3J = 7.4 Hz, ^4J = 1.7 Hz, 4 H), 1.32-1.62 (m, 5 H), 3.45-3.54 (m, 2 H), 5.62 (s, 2 H), 7.22-7.45 (m, 9 H), 7.47-7.60 (m, 1 H), 7.78-7.82 (m, 1 H), 7.95-8.00 (m, 3 H).

^{13}C NMR (75 MHz, acetone-d$_6$): δ 14.2 (CH_3), 20.1 (CH_2), 32.6 (CH_2), 35.9 (CH_2), 46.3 (CH_2), 49.7 (CH_2), 70.8 (CH), 111.5 (CH), 123.2 (CH), 123.8 (CH), 123.9 (CH), 125.6 (CH), 127.5 (CH), 127.9 (CH), 128.3 (CH), 128.5 (CH), 129.0 (C_{quat}), 129.5 (CH), 129.8 (CH), 136.4 (C_{quat}), 139.6 (C_{quat}), 141.0 (C_{quat}), 143.1 (C_{quat}), 162.5 (C_{quat}), 184.3 (C_{quat}).

MALDI-TOF (*m/z*): 430.3 ($C_{27}H_{27}NO_2S+H^+$).

IR $\tilde{\nu}$ [cm^{-1}]: 637 (m), 656 (m), 675 (m), 696 (m), 719 (m), 731 (m), 743 (m), 795 (m), 824 (m), 853 (m), 876 (m), 905 (m), 834 (w), 970 (w), 991 (w), 1018 (m), 1042 (m), 1065 (m), 1090 (m), 1157 (m), 1177 (m), 1194 (m), 1229 (m), 1265 (m), 1294 (m), 1304 (m), 1329 (m), 1356 (m), 1410 (m), 1450 (s), 1464 (s), 1510 (m), 1562 (m), 1599 (m), 1657 (w), 1713 (w), 2868 (w), 2930 (w), 2957 (w), 3030 (w), 3065 (w).

UV/Vis (C_3H_6O): λ_{max} (ε) = 386 (31100).

HRMS (ESI) calcd. for $C_{27}H_{27}NO_2S+H^+$: 430.1835; Found: 430.1842.

(*Z*)-2-(3-Benzylbenzo[*d*]thiazol-2(3*H*)-ylidene)-1-(4-((but-3-en-1-yloxy)methyl)phenyl)ethan-1-one (4c)

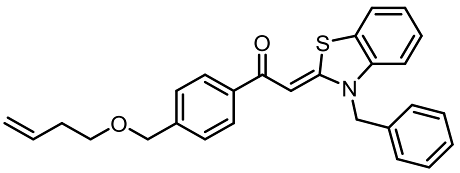

$C_{27}H_{25}NO_2S$

[427.16]

The synthesis was performed according to **GPIII** using *n*-hexane/acetone 3:1 as eluent for column chromatography to give 0.178 g (0.414 mmol, 41%) of **4c** as a yellow solid.

Mp: 153 °C.

R$_f$ (*n*-hexane/acetone 3:1): 0.35.

^{1}H NMR (600 MHz, acetone-d$_6$): δ 2.33-2.37 (m, 2 H), 3.53 (t, ^3J = 6.5 Hz, 2 H), 4.54 (s, 2 H), 4.74 (s, 3 H), 5.62 (s, 2 H), 6.94 (s, 1 H), 7.24-7.43 (m, 9 H), 7.50 (t, ^3J = 8.7 Hz, 1 H), 7.78-7.81 (m, 1 H), 7.95-8.00 (m, 2 H).

^{13}C NMR (150 MHz, acetone-d$_6$): δ 34.9 (CH), 46.3 (CH_2), 49.7 (CH_2), 70.4 (CH_2), 72.7 (CH_2), 88.3 (CH), 111.6 (CH), 116.5 (CH), 123.3 (CH), 123.9 (CH), 127.5 (CH), 127.6 (CH), 127.8 (C_{quat}), 127.91 (CH), 127.93 (CH), 128.3 (CH), 128.6 (CH), 129.5 (CH), 129.8 (CH), 136.3 (C_{quat}), 136.5 (C_{quat}), 141.0 (C_{quat}), 143.4 (C_{quat}), 162.8 (C_{quat}), 183.8 (C_{quat}).

MALDI-TOF (*m/z*): 428.3 ($C_{27}H_{25}NO_2S+H^+$).

IR $\tilde{\nu}$ [cm^{-1}]: 637 (m), 667 (m), 694 (m), 716 (s), 745 (s), 770 (m), 793 (m), 826 (m), 878 (m), 907 (m), 976 (w), 993 (w), 1016 (m), 1042 (m), 1065 (m), 1157 (w), 1177 (m), 1194 (m), 1229 (m), 1263 (m), 1302 (m), 1331 (m), 1356 (m), 1387 (m), 1412 (m), 1452 (s), 1472 (s), 1510 (m), 1558 (m), 1599 (m), 1661 (w), 1713 (w), 2853 (w), 2926 (w), 3030 (w), 3061 (w).

UV/Vis (C_3H_6O): λ_{max} (ε) = 387 (35300).

Anal calcd for $C_{27}H_{25}NO_2S$ [427.2]: C 75.85, H 5.89, N 3.28, S 7.50; Found: C 75.66, H 5.74, N 3.31, S 7.71.

(*Z*)-1-(4-((Allyloxy)methyl)phenyl)-2-(3-benzylbenzo[*d*]thiazol-2(3*H*)-ylidene)ethan-1-one (4d)

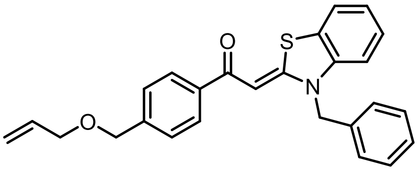

$C_{26}H_{23}NO_2S$

[413.14]

The synthesis was performed according to **GPIII** using *n*-hexane/acetone 3:1 to 1:1 as eluent for column chromatography to give 0.178 g (0.446 mmol, 45%) of **4d** as a yellow solid.

Mp: 130 °C.

R_f (*n*-hexane/acetone 3:1): 0.35.

^{1}H NMR (600 MHz, acetone-d$_6$): δ 4.03 (d, ^3J = 5.4 Hz, 2 H), 4.55 (s, 2 H), 4.74 (s, 3 H), 5.62 (s, 2 H), 6.94 (s, 1 H), 7.23-7.30 (m, 2 H), 7.32-7.43 (m, 7 H), 7.50 (d, ^3J = 7.5 Hz, 1 H), 7.79 (t, ^3J = 7.3 Hz, 1 H), 7.96-8.00 (m, 2 H).

^{13}C NMR (150 MHz, acetone-d$_6$): δ 46.3 (CH), 49.7 (CH_2), 71.7 (CH_2), 72.1 (CH_2), 88.3 (CH), 100.9 (CH), 111.6 (CH), 116.6 (CH), 123.2 (CH), 123.3 (CH), 123.9 (CH), 127.5 (CH), 127.6 (CH), 127.8 (C_{quat}), 128.0 (CH), 128.3 (CH), 128.6 (CH), 129.5 (CH), 129.8 (CH), 136.1 (C_{quat}), 136.3 (C_{quat}), 139.8 (C_{quat}), 141.0 (C_{quat}), 141.4 (C_{quat}), 162.5 (C_{quat}), 183.8 (C_{quat}).

MALDI-TOF (*m/z*): 414.3 ($C_{26}H_{23z}NO_2S$+H^+).

IR $\tilde{\nu}$ [cm^{-1}]: 625 (w), 637 (m), 644 (m), 694 (m), 716 (s), 745 (s), 793 (m), 824 (m), 837 (w), 853 (w), 878 (m), 907 (m), 922 (w), 976 (w), 993 (w), 1016 (m), 1041 (m), 1065 (m), 1090 (m), 1134 (w), 1157 (w), 1177 (m), 1192 (m), 1229 (m), 1263 (m), 1292 (m), 1302 (m), 1331 8m), 1356 (m), 1410 (m), 1452 (s), 1470 (s), 1510 (m), 1562 (m), 1567 (m), 1661 (w), 1713 (w), 2440 (w), 2848 (w), 2926 (w), 3028 (w), 3061 (w).

UV/Vis (C_3H_6O): λ_{max} (ε) = 386 (30700).

Anal calcd for $C_{26}H_{23}NO_2S$ [413.1]: C 75.52, H 5.61, N 3.39, S 7.75; Found: C 75.60, H 5.68, N 3.44, S 7.53.

(*Z*)-2-(3-Benzylbenzo[*d*]thiazol-2(3*H*)-ylidene)-1-(4-((benzyloxy)methyl)phenyl)ethan-1-one (4e)

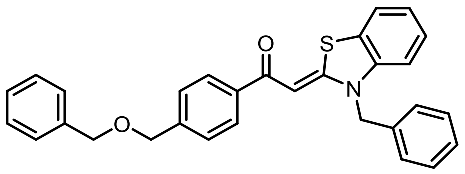

$C_{30}H_{25}NO_2S$

[463.16]

The synthesis was performed according to **GPIII** using *n*-hexane/acetone 3:1 to 1:1 as eluent for column chromatography to give 0.116 g (0.250 mmol, 25%) of **4e** as a red resin.

R_f (*n*-hexane/acetone 3:1): 0.28.

^{1}H NMR (300 MHz, acetone-d$_6$): δ 4.04 (s, 2 H), 4.49 (s, 4 H), 7.05-7.11 (m, 3 H), 7.15-7.24 (m, 16 H).

^{13}C NMR (75 MHz, acetone-d$_6$): δ 49.7 (CH_2), 63.6 (CH_2), 64.7 (CH_2), 88.3 (CH), 106.9 (CH), 110.1 (CH), 111.6 (CH), 114.2 (CH), 127.3 (CH), 127.6 (CH), 127.9 (CH), 128.2 (CH), 128.3 (C_{quat}), 128.4 (CH), 128.6 (CH), 128.9 (CH), 129.0 (CH), 129.8 (CH), 136.6 (C_{quat}), 138.4 (C_{quat}), 140.3 (C_{quat}), 143.4 (C_{quat}), 153.2 (C_{quat}), 163.2 (C_{quat}), 182.1 (C_{quat}).

MALDI-TOF (*m/z*): 464.3 ($C_{30}H_{25}NO_2S+H^+$).

IR $\tilde{\nu}$ [cm^{-1}]: 650 (m), 696 (s), 733 (s), 804 (m), 814 (m), 847 (w), 878 (w), 910 (m), 1011 (m), 1036 (m), 1080 (m), 1103 (w), 1157 (w), 1177 (w), 1206 (m), 1273 (m), 1310 (w), 1329 (w), 1368 (w), 1391 (w), 1402 (w), 1414 (w), 1452 (m), 1497 (m), 1585 (w), 1607 (w), 1807 (w), 1950 (w), 2872 (w), 2914 (w), 2932 (w), 3030 (w), 3063 (w), 3088 (w), 3111 (w).

UV/Vis (C_3H_6O): λ_{max} (ε) = 385 (1600).

HRMS (ESI) calcd. for $C_{30}H_{25}NO_2S+H^+$: 464.1679; Found: 464.1680.

(*Z*)-2-(3-Benzylbenzo[*d*]thiazol-2(3*H*)-ylidene)-1-(4-(isopropoxymethyl)phenyl)ethan-1-one (4f)

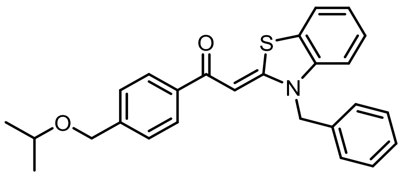

$C_{26}H_{25}NO_2S$

[415.17]

The synthesis was performed according to **GPIII** using *n*-hexane/acetone 3:1 to 2:1 to 1:1 as eluent for column chromatography to give 0.262 g (0.648 mmol, 65%) of **4f** as a yellow solid.

Mp: 157 °C.

R_f (*n*-hexane/acetone 3:1): 0.38.

^{1}H NMR (300 MHz, acetone-d$_6$/CS$_2$ 5:1): δ 1.19 (d, ^3J = 6.1 Hz, 6 H), 4.54 (s, 1 H), 4.71 (s, 2 H), 5.58 (s, 2 H), 6.86 (s, 1 H), 7.23-7.38 (m, 9 H), 7.45-7.49 (m, 1 H), 7.73-7.78 (m, 1 H), 7.89-7.96 (m, 2 H).

^{13}C NMR (75 MHz, acetone-d$_6$/CS$_2$ 5:1): δ 22.5 (CH_3), 46.4 (CH), 49.7 (CH_2), 70.0 (CH_2), 71.7 (CH), 88.3 (CH), 111.5 (CH), 123.2 (CH), 123.8 (CH), 127.5 (CH), 127.6 (CH), 127.9 (CH), 128.0 (C_{quat}), 128.3 (CH), 128.52 (CH), 128.54 (CH), 129.4 (CH), 129.7 (CH), 136.1 (C_{quat}), 140.4 (C_{quat}), 140.9 (C_{quat}), 141.1 (C_{quat}), 143.4 (C_{quat}), 162.3 (C_{quat}), 162.6 (C_{quat}), 183.8 (C_{quat}).

EI + MS (70 eV, *m/z* (%)): 415 ($[C_{26}H_{25}NO_2S]^+$, 39), 398 (22), 391 (18), 239 (20), 253 ($[C_{16}H_{15}NS]^+$, 18), 238 ($[C_{15}H_{12}NS]^+$, 94), 236 (31), 224 (18), 238 ($[C_{14}H_9NS]^+$, 33), 177 ($[C_{11}H_{13}O_2]^+$, 43), 153 (21), 104 ($[C_8H_8]^+$, 66), 91 ($[C_7H_7]^+$, 100), 89 (11), 65 ($[C_5H_5]^+$, 13).

IR $\tilde{\nu}$ [cm^{-1}]: 646 (m), 664 (m), 694 (m), 718 (s), 745 (s), 754 (m), 772 (m), 793 (m), 826 (m), 860 (m), 878 (m), 907 (m), 993 (w), 1016 (m), 1043 (m), 1065 (m), 1090 (m), 1107 (w), 1126 (m), 1157 (w), 1180 (m), 1194 (m), 1229 (m), 1263 (m), 1292 (w), 1302 (m), 1331 (m), 1339 (m), 1379 (m), 1412 (m), 1452 (s), 1470 (s), 1485 (s), 1560 (m), 1597 (m), 2864 (w), 2968 (w), 3028 (w), 3059 (w).

UV/Vis (C_3H_6O): λ_{max} (ε) = 386 (41300).

Anal calcd for $C_{26}H_{25}NO_2S$ [415.2]: C 75.15, H 6.06, N 3.37, S 7.72; Found: C 75.35, H 6.18, N 3.39, S 7.79.

(*Z*)-2-(3-Benzylbenzo[*d*]thiazol-2(3*H*)-ylidene)-1-(4-(ethoxymethyl)phenyl)ethan-1-one (4g)

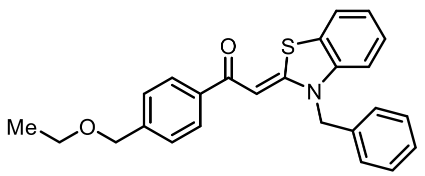

$C_{25}H_{23}NO_2S$

[401.14]

The synthesis was performed according to **GPIII** using *n*-hexane/acetone 4:1 to 2:1 as eluent for column chromatography to give 0.193 g (0.481 mmol, 48%) of **4g** as a yellow solid.

Mp: 131 °C.

R_f (*n*-hexane/acetone 3:1): 0.30.

^{1}H NMR (300 MHz, acetone-d$_6$/CS$_2$ 5:1): δ 1.18 (t, ^3J = 7.0 Hz, 3 H), 3.52 (q, ^3J = 7.0 Hz, 2 H), 4.52 (s, 2 H), 5.62 (s, 2 H), 7.22-7.42 (m, 11 H), 7.77-7.81 (m, 1 H), 7.94-8.01 (m, 2 H).

^{13}C NMR (75 MHz, acetone-d$_6$/CS$_2$ 5:1): δ 15.5 (CH_3), 49.7 (CH_2), 66.3 (CH_2), 72.5 (CH_2), 111.5 (CH), 123.2 (CH), 123.8 (CH), 127.5 (CH), 127.86 (CH), 127.92 (CH), 128.3 (C_{quat}), 128.5 (CH), 129.5 (CH), 129.8 (CH), 136.4 (C_{quat}), 139.6 (C_{quat}), 141.0 (C_{quat}), 143.0 (C_{quat}), 162.5 (C_{quat}), 184.3 (C_{quat}).

MALDI-TOF (*m/z*): 402.3 ($C_{25}H_{23}NO_2S+H^+$).

IR $\tilde{\nu}$ [cm^{-1}]: 627 (w), 662 (w), 692 (m), 708 (m), 733 (m), 743 (s), 756 (m), 799 (w), 826 (m), 849 (m), 876 8m), 893 (w), 908 (m), 935 (w), 972 (w), 1001 (w), 1018 (m), 1045 (m), 1067 (m), 1092 (m), 1111 (m), 1134 (w), 1159 (w), 1177 (m), 1202 (m), 1229 (m), 1271 (m), 1300 (m), 1331 (m), 1344 (m), 1371 (w), 1398 (w), 1416 (m), 1452 (s), 1470 (s), 1510 (w), 1557 (m), 1601 (m), 1672 (w), 1713 (w), 2386 (w), 2791 (w), 2874 (w), 2926 (w), 2968 (w), 3061 (w).

UV/Vis (C_3H_6O): λ_{max} (ε) = 385 (39000).

Anal calcd for $C_{25}H_{23}NO_2S$ [401.1]: C 74.78, H 5.77, N 3.49, S 7.98; Found: C 74.60, H 5.68, N 3.47, S 7.98.

(*Z*)-2-(3-Benzylbenzo[*d*]thiazol-2(3*H*)-ylidene)-1-(4-(methoxymethyl)phenyl)ethan-1-one (4h)

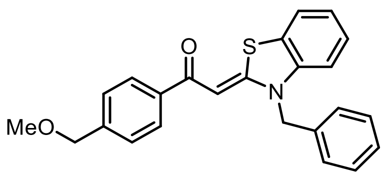

$C_{24}H_{21}NO_2S$

[387.13]

The synthesis was performed according to **GPIII** using *n*-hexane/acetone 3:1 to 1:1 as eluent for column chromatography to give 0.115 g (0.297 mmol, 30%) of **4h** as a yellow solid.

Mp: 111 °C.

R_f (*n*-hexane/acetone 3:1): 0.30.

^{1}H NMR (600 MHz, acetone-d$_6$): δ 3.34 (s, 3 H), 4.47 (s, 2 H), 5.69 (s, 2 H), 6.91-6.95 (m, 1 H), 7.21-7.47 (m, 9 H), 7.50-7.60 (m, 1 H), 7.80-7.82 (m, 1 H), 7.96-8.00 (m, 2 H).

^{13}C NMR (150 MHz, acetone-d$_6$): δ 49.7 (CH_2), 58.2 (CH_3), 74.5 (CH_2), 88.2 (CH), 111.6 (CH), 123.2 (CH), 123.8 (CH), 126.8 (C_{quat}), 127.5 (CH), 127.6 (CH), 127.96 (CH), 127.98 (CH), 128.6 (CH), 129.0 (CH), 129.5 (CH), 129.8 (CH), 130.1 (CH), 130.4 (CH), 131.1 (C_{quat}), 136.4 (C_{quat}), 139.8 (C_{quat}), 141.1 (C_{quat}), 142.6 (C_{quat}), 144.5 (C_{quat}), 162.6 (C_{quat}), 165.0 (C_{quat}), 184.3 (C_{quat}).

MALDI-TOF (*m/z*): 388.3 ($C_{24}H_{21}NO_2S+H^+$).

IR $\tilde{\nu}$ [cm^{-1}]: 617 (m), 635 (m), 698 (m), 721 (m), 733 (m), 797 (w), 824 (m), 853 (m), 876 (m), 905 (m), 968 (m), 1003 (w), 1018 (m), 1042 (m), 1065 (m), 1090 (m), 1138 (w), 1159 (w), 1177 (m), 1192 (m), 1229 (m), 1279 (m), 1329 (m), 1356 (m), 1393 (m), 1410 (m), 1450 (s), 1464 (s), 1510 (w), 1562 (m), 1599 (m), 1651 (m) ,1659 (m). 1717 (m), 2822 (w), 2849 (w), 2891 (w), 2926 (w), 2980 (w), 3030 (w), 3061 (w).

UV/Vis (C_3H_6O): λ_{max} (ε) = 385 (15500).

Anal calcd for $C_{24}H_{21}NO_2S$ [387.1]: C 74.39, H 5.46, N 3.61, S 8.27; Found: C 74.29, H 5.18, N 3.33, S 8.13.

(*Z*)-2-(3-Benzylbenzo[*d*]thiazol-2(3*H*)-ylidene)-1-(4-(phenoxymethyl)phenyl)ethan-1-one (4i)

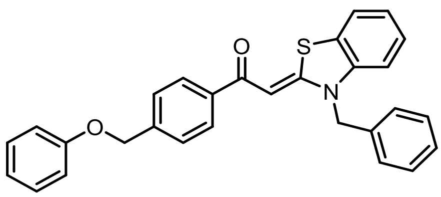

$C_{29}H_{23}NO_2S$

[449.14]

The synthesis was performed according to **GPIII** using *n*-hexane/acetone 3:1 as eluent for column chromatography to give 0.163 g (0.363 mmol, 36%) of **4i** as an orange resin.

R*f* (*n*-hexane/acetone 3:1): 0.29.

^{1}H NMR (300 MHz, acetone-d$_6$): δ 4.44 (s, 2 H), 5.49 (s, 2 H), 6.76-6.85 (m, 2 H), 7.08 (td, ^3J = 7.7 Hz, ^4J = 1.5 Hz, 1 H), 7.15-7.19 (m, 2 H), 7.21-7.37 (m, 7 H), 7.44-7.50 (m, 2 H), 7.58 (dd, ^3J = 8.0 Hz, ^4J = 1.4 Hz, 1 H), 7.69 (d, ^3J = 8.4 Hz, 2 H), 8.12-8.16 (m, 2 H).

^{13}C NMR (75 MHz, acetone-d$_6$): δ 36.1 (CH$_2$), 51.2 (CH$_2$), 122.7 (CH), 126.7 (CH), 126.8 (CH), 127.5 (C$_{quat}$), 128.1 (CH), 128.6 (CH), 129.0 (CH), 129.6 (CH), 129.7 (CH), 129.8 (CH), 130.25 (CH), 130.29 (CH), 131.0 (CH), 131.1 (CH), 137.2 (C$_{quat}$), 138.9 (C$_{quat}$), 140.9 (C$_{quat}$), 144.6 (C$_{quat}$), 152.1 (C$_{quat}$), 165.2 (C$_{quat}$), 170.2 (C$_{quat}$), 187.0 (C$_{quat}$).

MALDI-TOF (*m/z*): 450.3 ($C_{29}H_{23}NO_2S+H^+$).

IR $\tilde{\nu}$ [cm^{-1}]: 613 (m), 635 (m), 662 (w), 689 (s), 731 (s), 824 (m), 856 (m), 878 (m), 907 (m), 914 (m), 972 (m), 1001 (m), 1018 (m), 1040 (w), 1069 (s), 1111 (w), 1130 (w), 1161 (m), 1177 (s), 1192 (s), 1263 (s), 1294 (m), 1358 (m), 1391 (m), 1414 (m), 1435 (m), 1454 (m), 1470 (m), 1558 (m), 1582 (m), 1607 (m), 1651 (m), 1659 (m), 1732 (m), 2363 (w), 2857 (w), 2926 (w), 2953 (w), 3007 (w), 3030 (w), 3061 (w), 3501 (w).

UV/Vis (C_3H_6O): λ_{max} (ε) = 385 (4600).

HRMS (ESI) calcd. for $C_{29}H_{23}NO_2S+H^+$: 449.1449; Found: 449.1444.

(*Z*)-2-(3-Benzylbenzo[*d*]thiazol-2(3*H*)-ylidene)-1-(4-(hydroxymethyl)phenyl)ethan-1-one (4j)

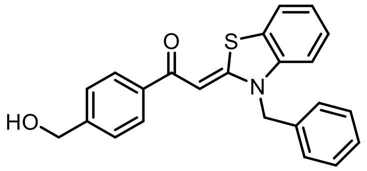

$C_{23}H_{19}NO_2S$

[373.11]

The synthesis was performed according to **GPIII** using *n*-hexane/acetone 3:1 to 2:1 to 1:1 to pure acetone as eluent for column chromatography to give 0.128 g (0.343 mmol, 34%) of **4j** as a red solid.

Mp: 102 °C.

R_f (*n*-hexane/acetone 3:1): 0.21.

^{1}H NMR (300 MHz, acetone-d$_6$): δ 3.58 (s, 2 H), 4.67 (s, 1 H), 5.61 (s, 2 H), 6.92 (s, 1 H), 7.03-7.56 (m, 10 H), 7.78 (d, ^3J = 7.9 Hz, 1 H), 7.90-7.97 (m, 2 H).

^{13}C NMR (75 MHz, acetone-d$_6$): δ 49.6 (CH_2), 67.6 (CH_2), 88.2 (CH), 111.5 (CH), 123.2 (CH), 123.8 (CH), 127.0 (CH), 127.5 (CH), 127.9 (CH), 128.2 (C_{quat}), 128.5 (CH), 129.0 (CH), 129.8 (CH), 136.4 (C_{quat}), 139.2 (C_{quat}), 141.0 (C_{quat}), 146.4 (C_{quat}), 162.4 (C_{quat}), 184.4 (C_{quat}).

MALDI-TOF (*m/z*): 374.3 ($C_{23}H_{19}NO_2S+H^+$).

IR $\tilde{\nu}$ [cm^{-1}]: 694 (m), 721 (m), 733 (s), 743 (s), 781 (w), 806 (w), 818 (m), 876 (m), 905 (m), 989 (w), 1001 (m), 1016 (m), 1040 (m), 1065 (m), 1088 (m), 1119 (m), 1136 (m), 1157 (m), 1179 (m), 1229 (m), 1261 (w), 1304 (m), 1329 (m), 1354 (m), 1383 (m), 1393 (m), 1408 (w), 1449 (s), 1464 (s), 1512 (m), 1557 (m), 1597 (m), 2359 (w), 2966 (w), 3057 (w).

UV/Vis (C_3H_6O): λ_{max} (ε) = 256 (15300), 385 (22400).

Anal calcd for $C_{23}H_{19}NO_2S$ [373.1]: C 73.97, H 5.13, N 3.75, S 8.57; Found: C 73.80, H 5.08, N 3.45, S 8.28.

3.2 NMR spectra of etherified aroyl-*S,N*-ketene acetals 4

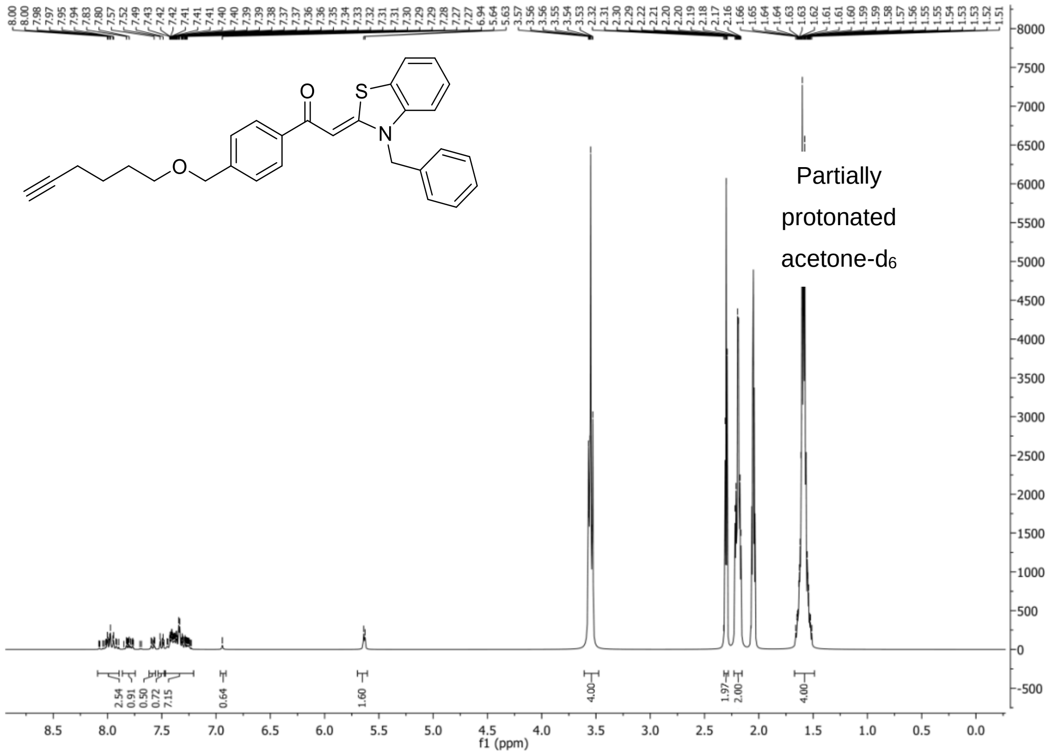

Figure S1. ^{1}H NMR spectrum (*Z*)-2-(3-benzylbenzo[*d*]thiazol-2(3*H*)-ylidene)-1-(4-((hex-5-yn-1-yloxy)methyl)phenyl)ethan-1-one (**4a**) (300 MHz, acetone-d_6, 293 K).

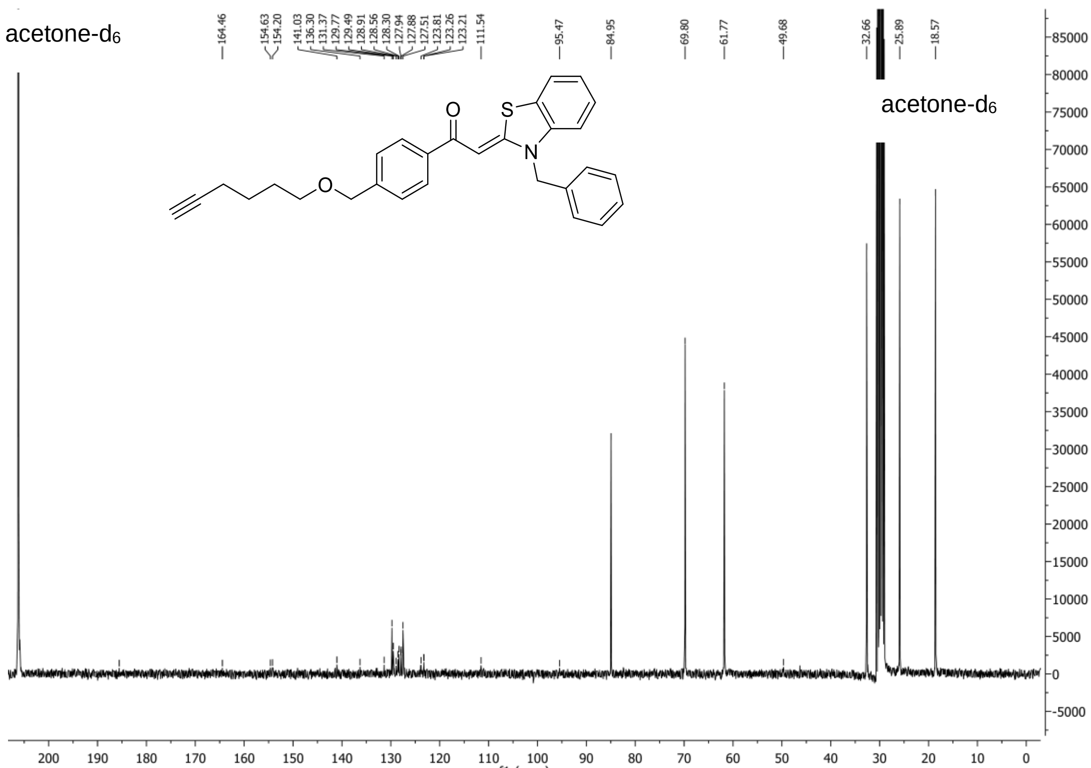

Figure S2. ^{13}C NMR spectrum (*Z*)-2-(3-benzylbenzo[*d*]thiazol-2(3*H*)-ylidene)-1-(4-((hex-5-yn-1-yloxy)methyl)phenyl)ethan-1-one (**4a**) (75 MHz, acetone-d_6, 293 K).

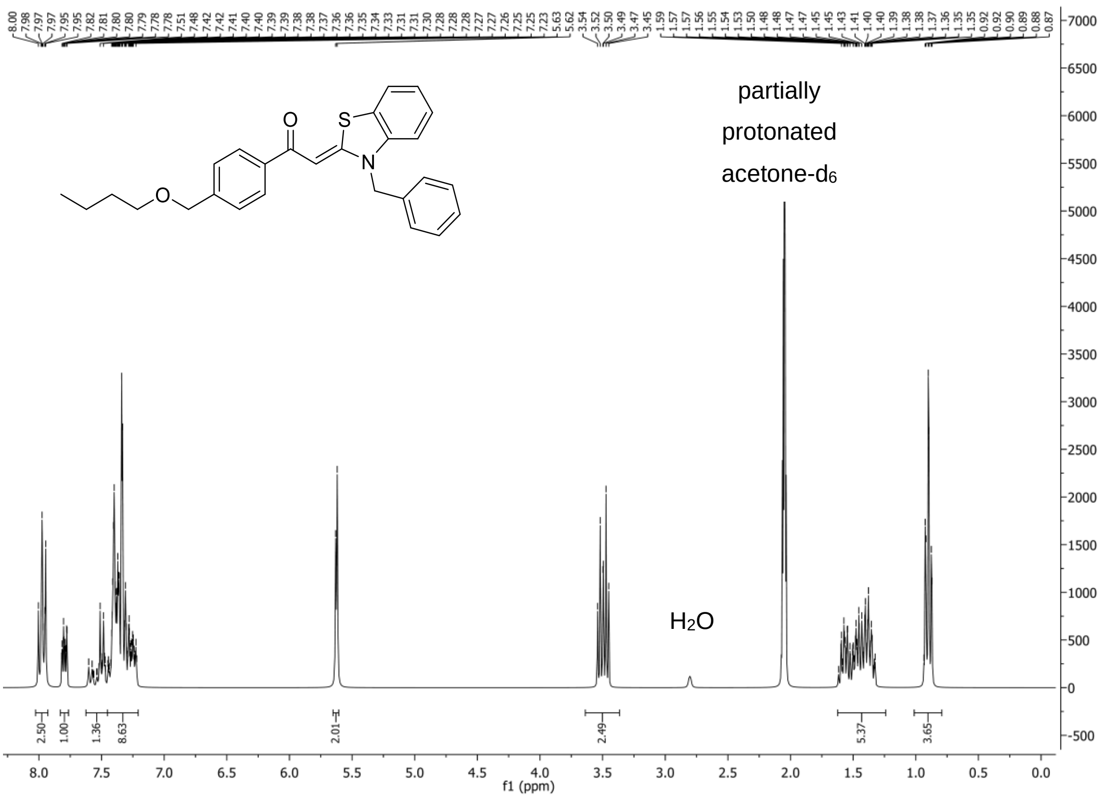

Figure S3. ^{1}H NMR spectrum (*Z*)-2-(3-benzylbenzo[*d*]thiazol-2(3*H*)-ylidene)-1-(4-(butoxymethyl)phenyl)ethan-1-one (**4b**) (300 MHz, acetone-d$_6$, 293 K).

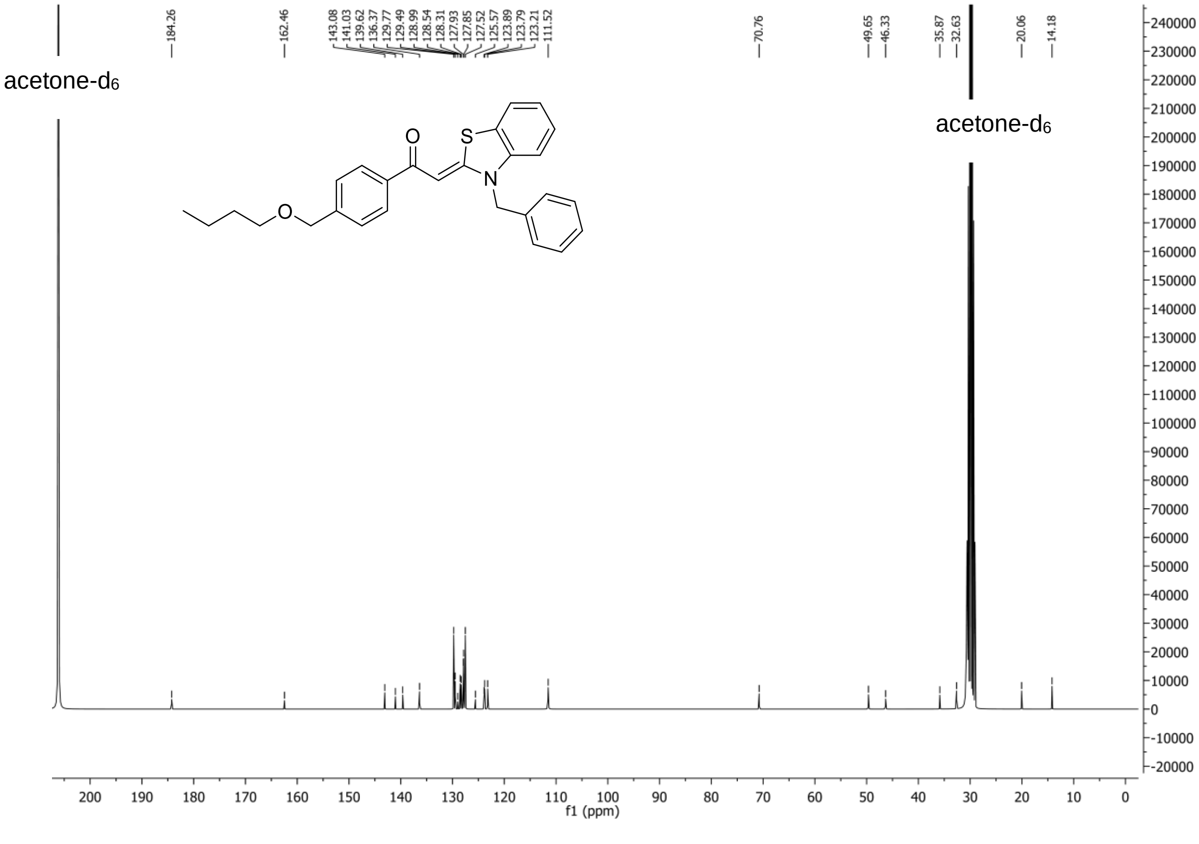

Figure S4. ^{13}C NMR spectrum (*Z*)-2-(3-benzylbenzo[*d*]thiazol-2(3*H*)-ylidene)-1-(4-(butoxymethyl)phenyl)ethan-1-one (**4b**) (75 MHz, acetone-d$_6$, 293 K).

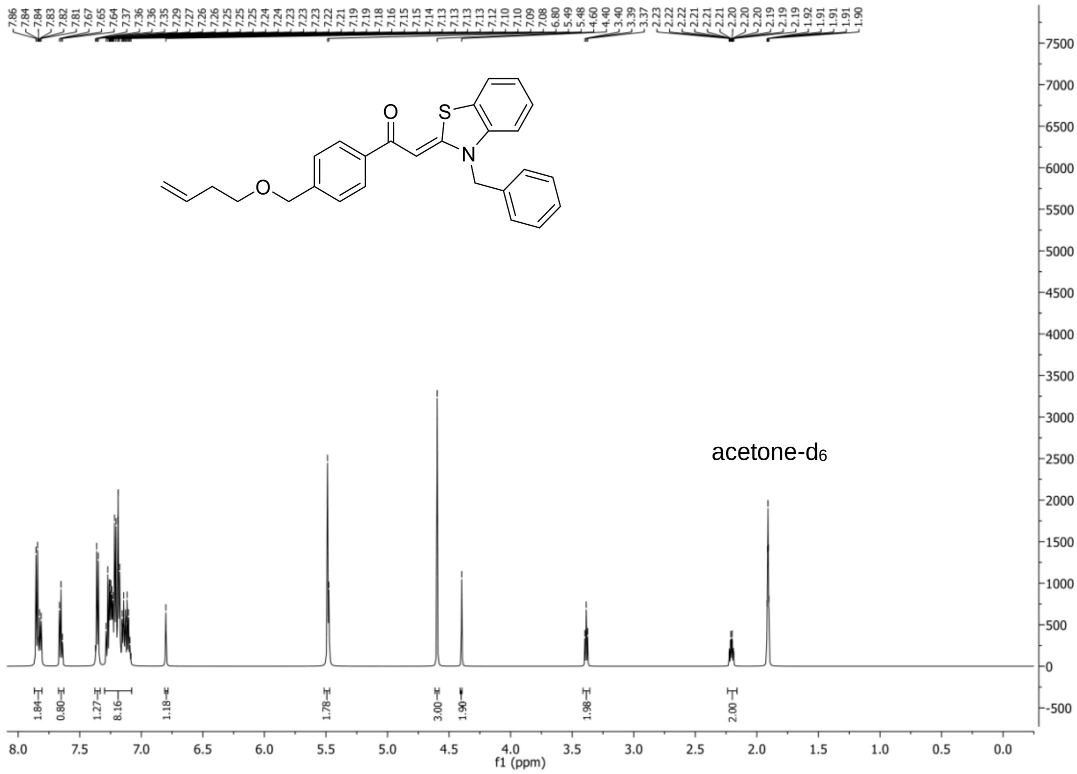

Figure S5. ^{1}H NMR spectrum (*Z*)-2-(3-benzylbenzo[*d*]thiazol-2(3*H*)-ylidene)-1-(4-((but-3-en-1-yloxy)methyl)phenyl)ethan-1-one (**4c**) (600 MHz, acetone-d_6, 293 K).

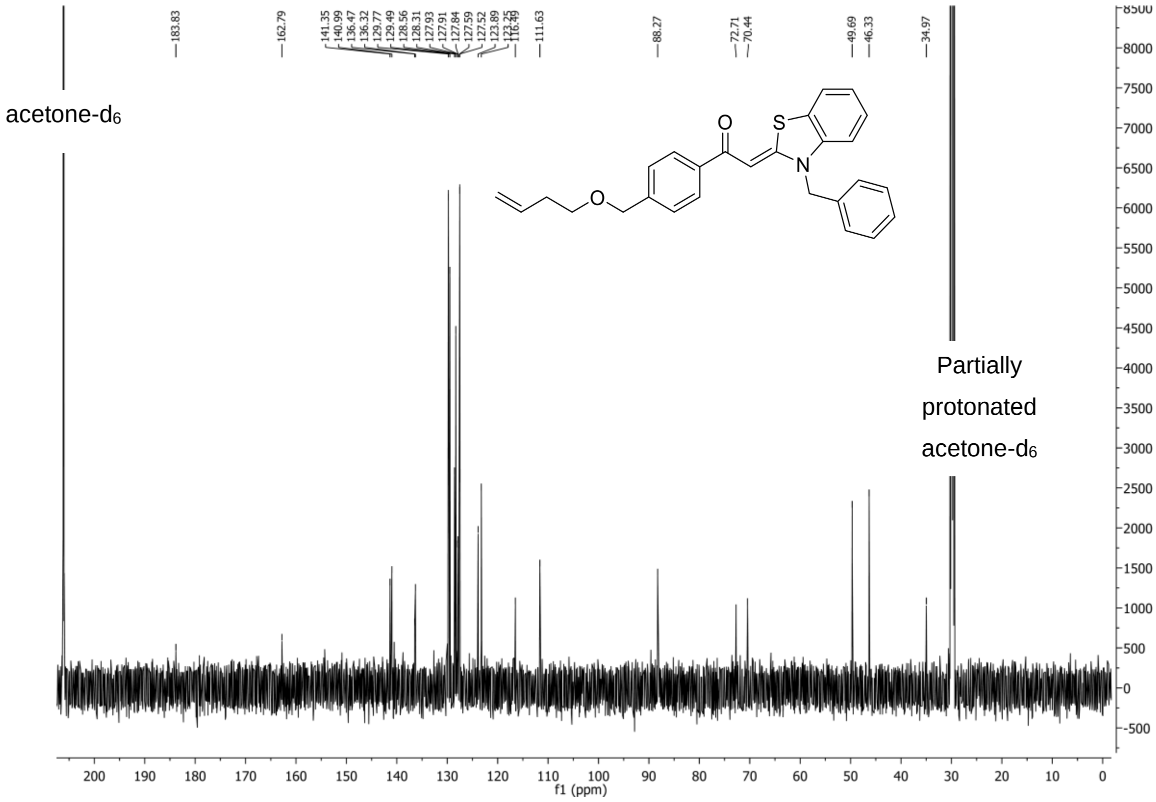

Figure S6. ^{13}C NMR spectrum (*Z*)-2-(3-benzylbenzo[*d*]thiazol-2(3*H*)-ylidene)-1-(4-((but-3-en-1-yloxy)methyl)phenyl)ethan-1-one (**4c**) (150 MHz, acetone-d_6, 293 K).

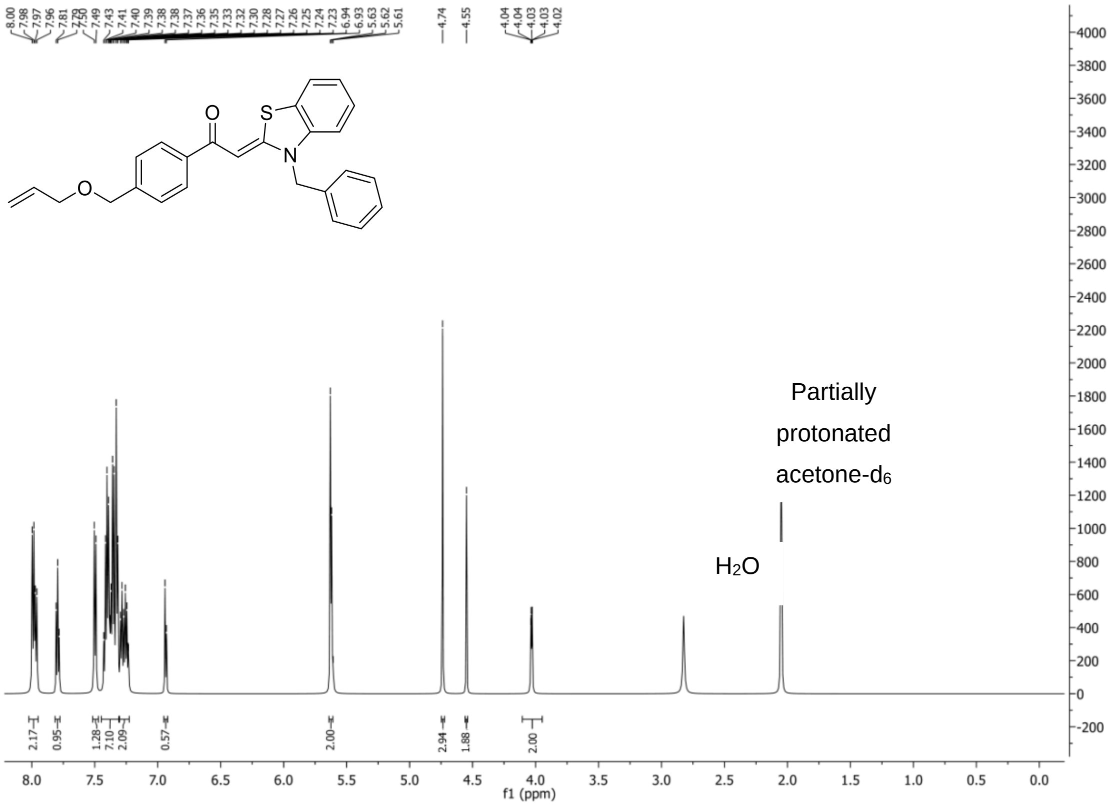

Figure S7. ^{1}H NMR spectrum (*Z*)-1-(4-((allyloxy)methyl)phenyl)-2-(3-benzylbenzo[*d*]thiazol-2(3*H*)-ylidene)ethan-1-one (**4d**) (600 MHz, acetone-d$_6$, 293 K).

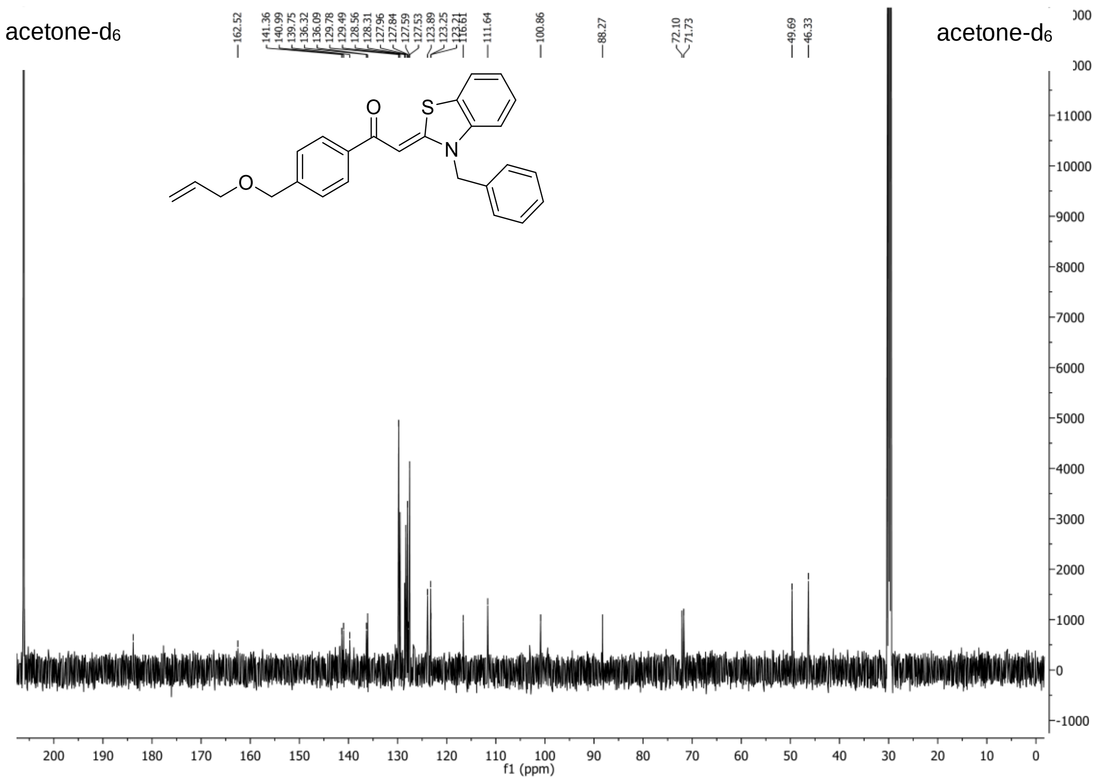

Figure S8. ^{13}C NMR spectrum (*Z*)-1-(4-((allyloxy)methyl)phenyl)-2-(3-benzylbenzo[*d*]thiazol-2(3*H*)-ylidene)ethan-1-one (**4d**) (150 MHz, acetone-d$_6$, 293 K).

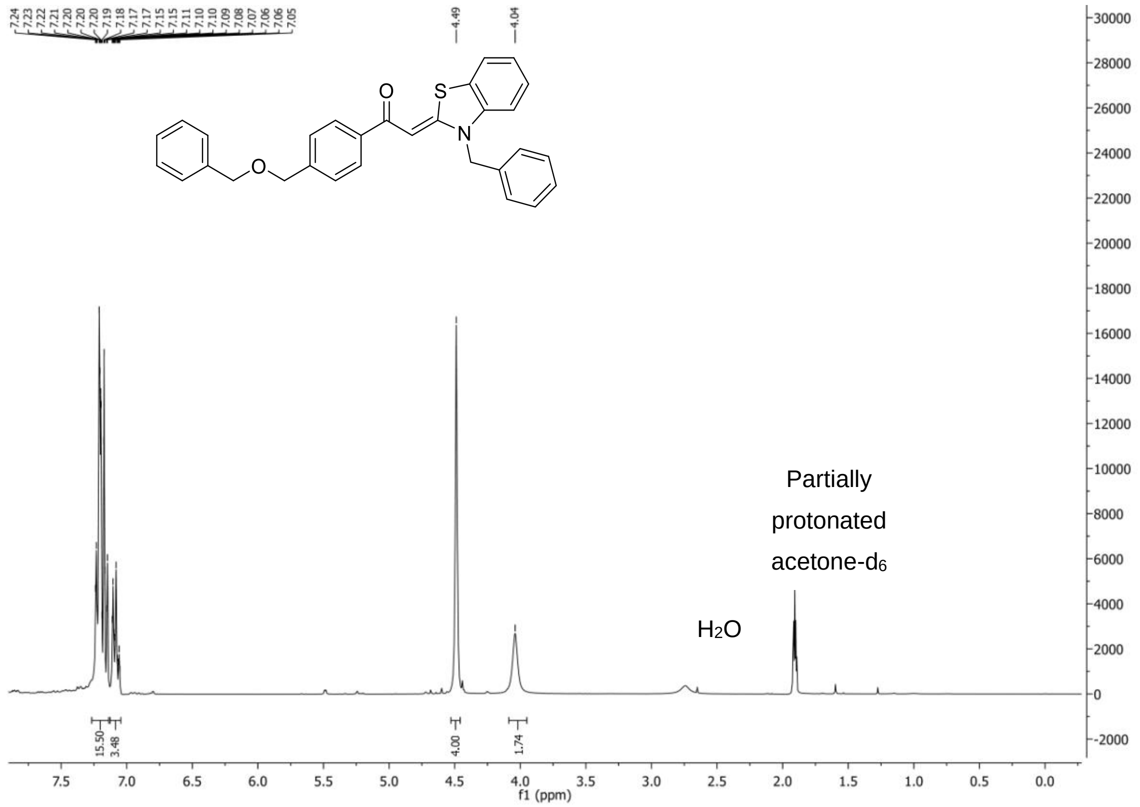

Figure S9. ^{1}H NMR spectrum (*Z*)-2-(3-benzylbenzo[*d*]thiazol-2(3*H*)-ylidene)-1-(4-((benzyloxy)methyl)phenyl)ethan-1-one (**4e**) (300 MHz, acetone-d$_6$, 293 K).

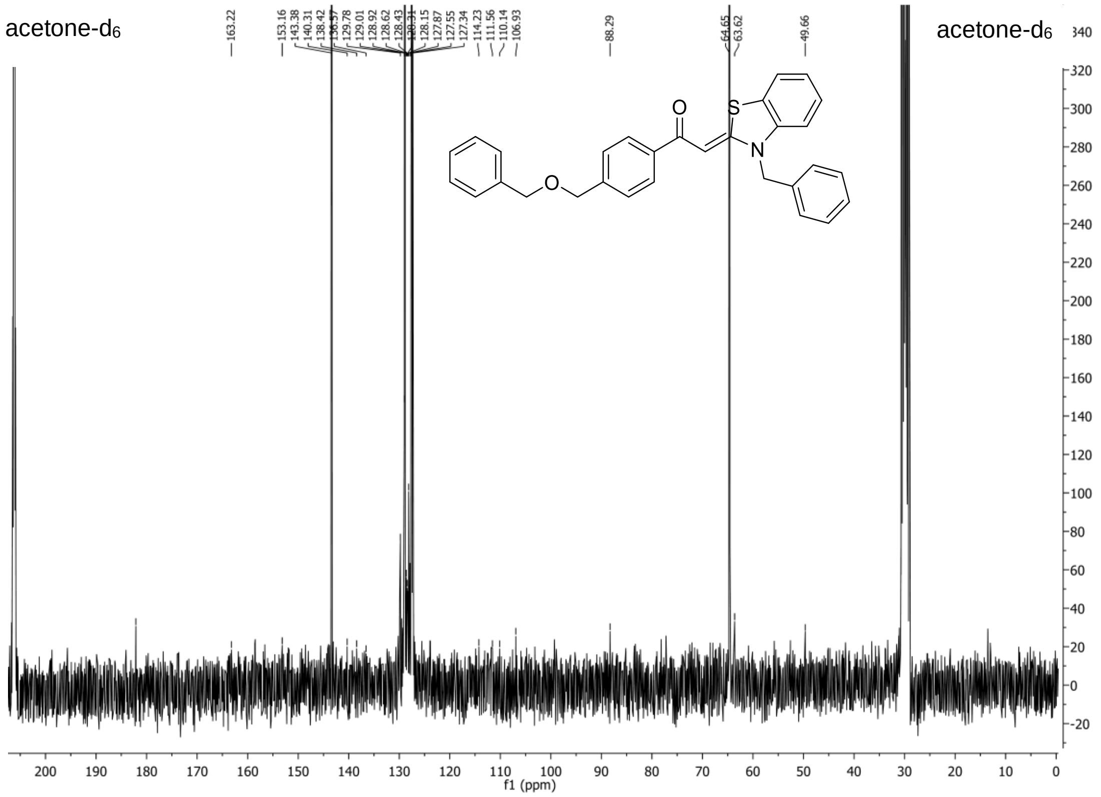

Figure S10. ^{13}C NMR spectrum (*Z*)-2-(3-benzylbenzo[*d*]thiazol-2(3*H*)-ylidene)-1-(4-((benzyloxy)methyl)phenyl)ethan-1-one (**4e**) (75 MHz, acetone-d$_6$, 293 K).

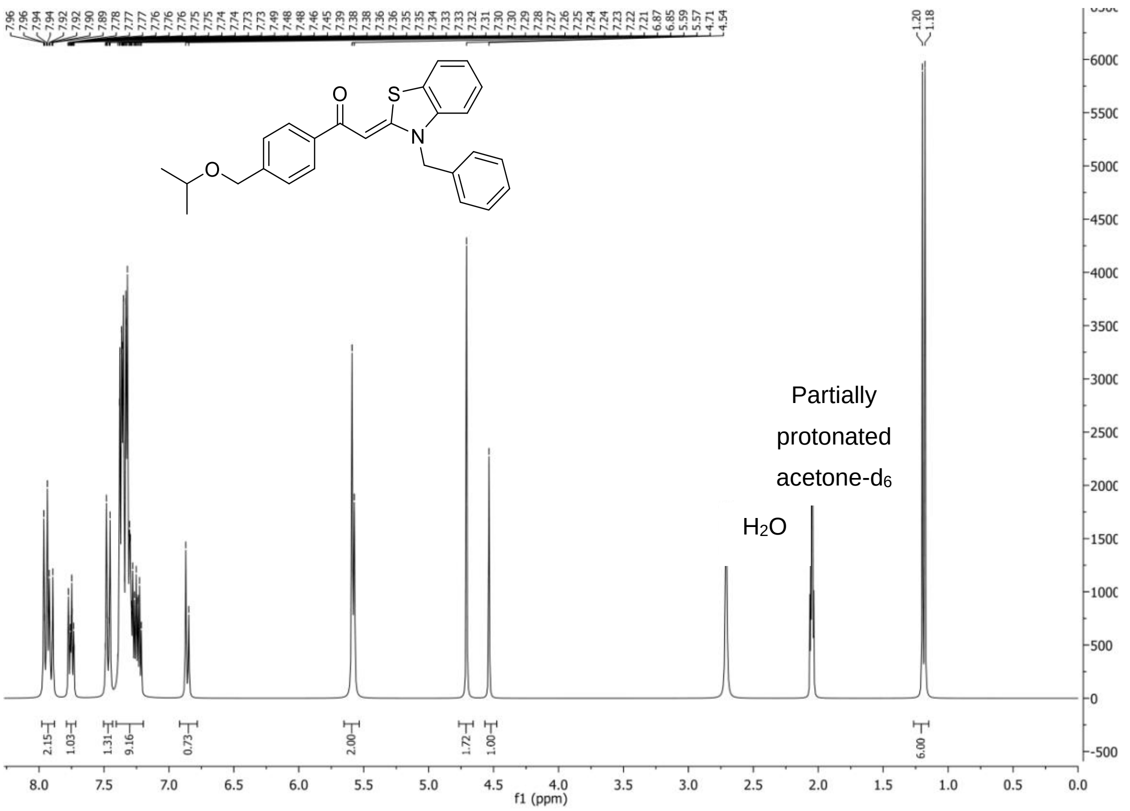

Figure S11. ^{1}H NMR spectrum (*Z*)-2-(3-benzylbenzo[*d*]thiazol-2(3*H*)-ylidene)-1-(4-(isopropoxymethyl)phenyl)ethan-1-one (**4f**) (300 MHz, acetone-d_6/CS_2 5:1, 293 K).

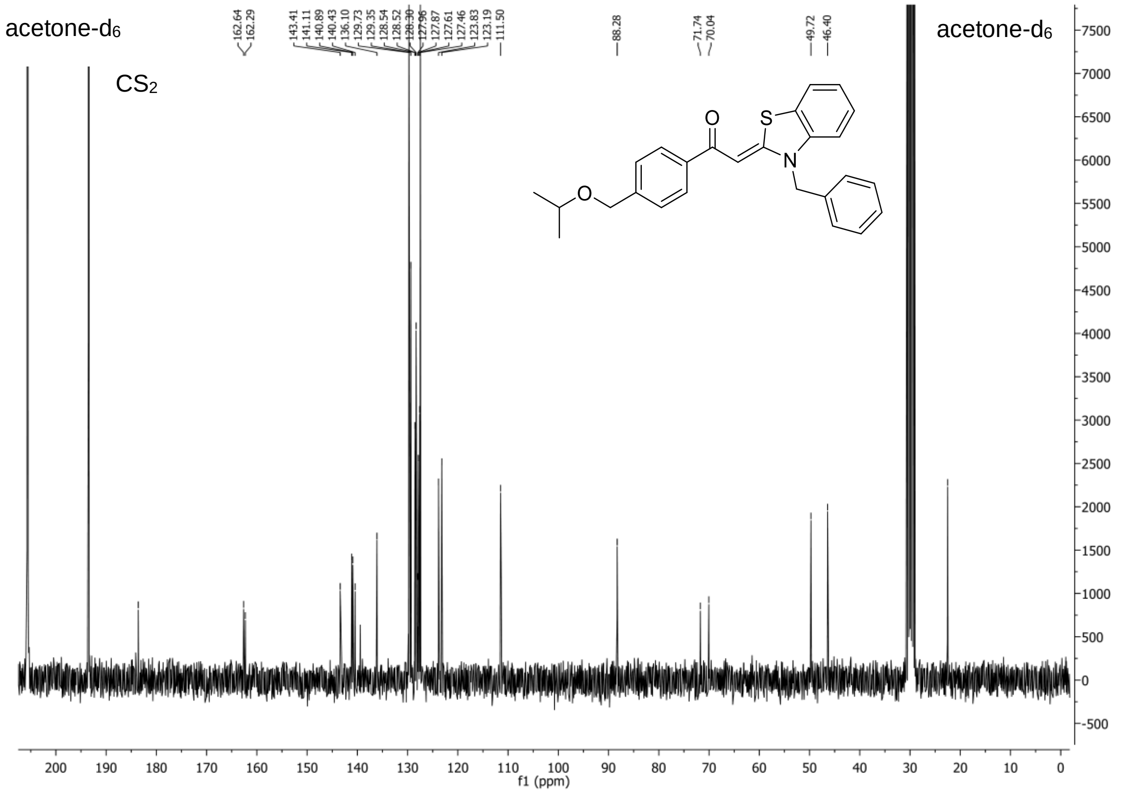

Figure S12. ^{13}C NMR spectrum (*Z*)-2-(3-benzylbenzo[*d*]thiazol-2(3*H*)-ylidene)-1-(4-(isopropoxymethyl)phenyl)ethan-1-one (**4f**) (75 MHz, acetone-d_6/CS_2 5:1, 293 K).

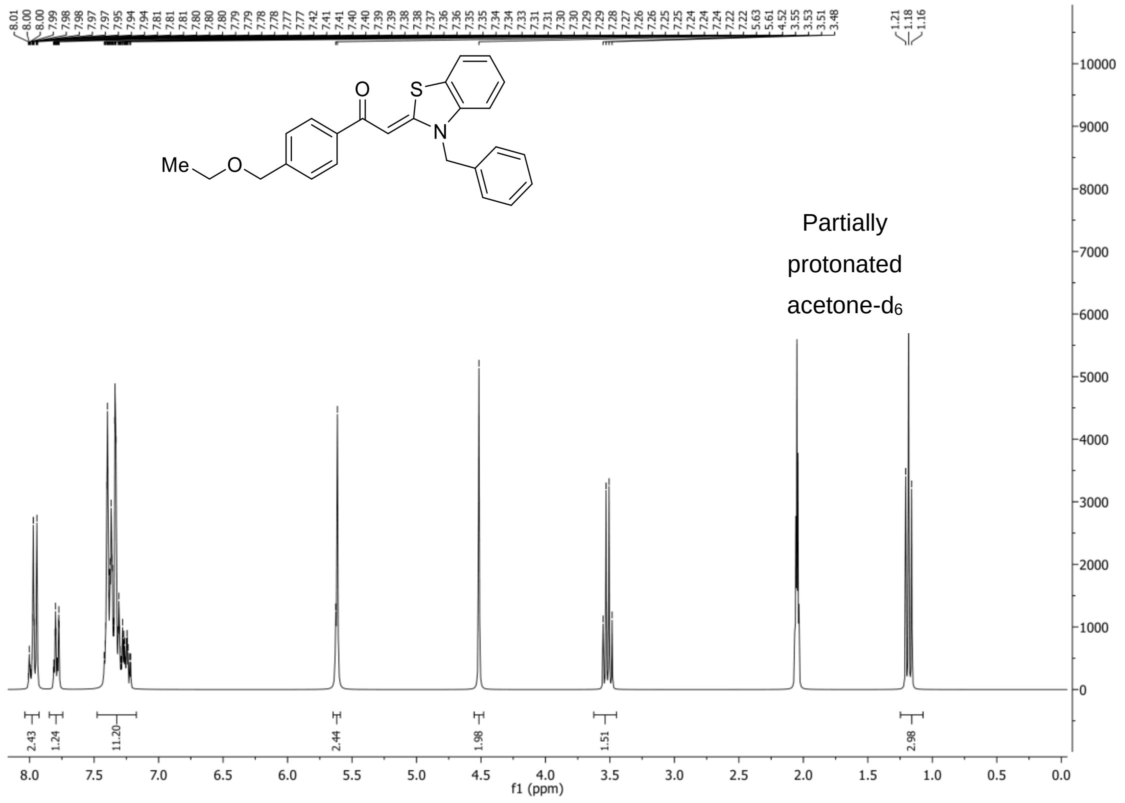

Figure S13. ^{1}H NMR spectrum (*Z*)-2-(3-benzylbenzo[*d*]thiazol-2(3*H*)-ylidene)-1-(4-(ethoxymethyl)phenyl)ethan-1-one (**4g**) (300 MHz, acetone-d_6, 293 K).

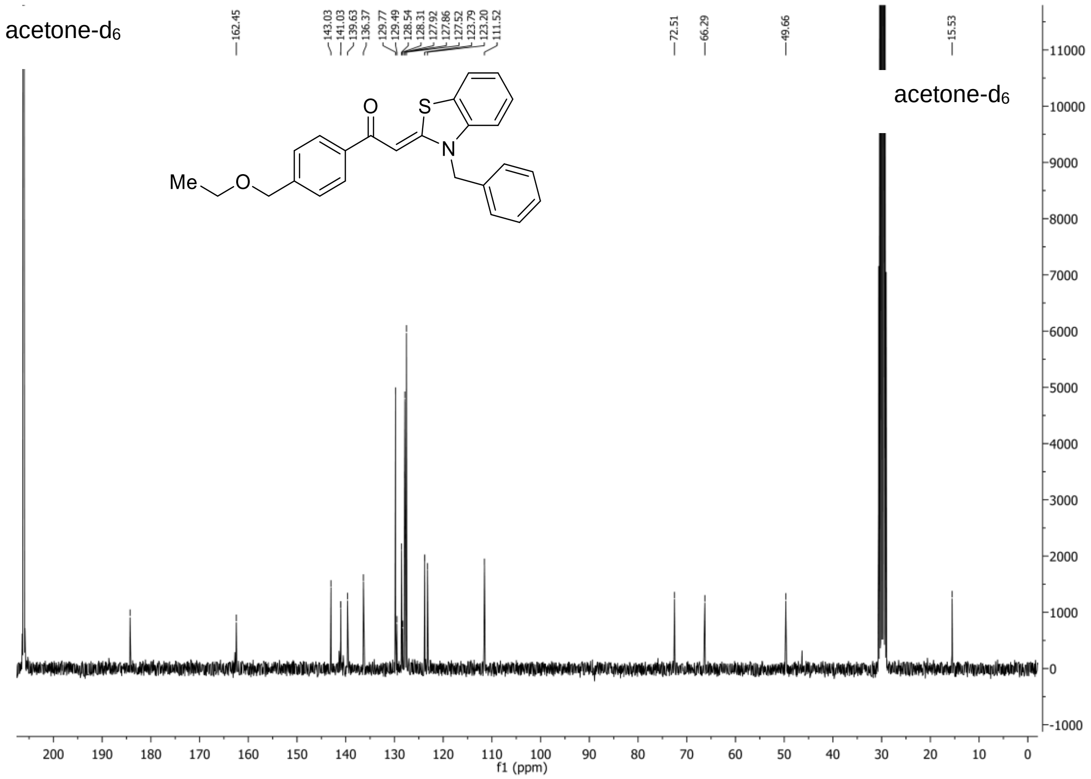

Figure S14. ^{13}C NMR spectrum (*Z*)-2-(3-benzylbenzo[*d*]thiazol-2(3*H*)-ylidene)-1-(4-(ethoxymethyl)phenyl)ethan-1-one (**4g**) (75 MHz, acetone-d_6, 293 K).

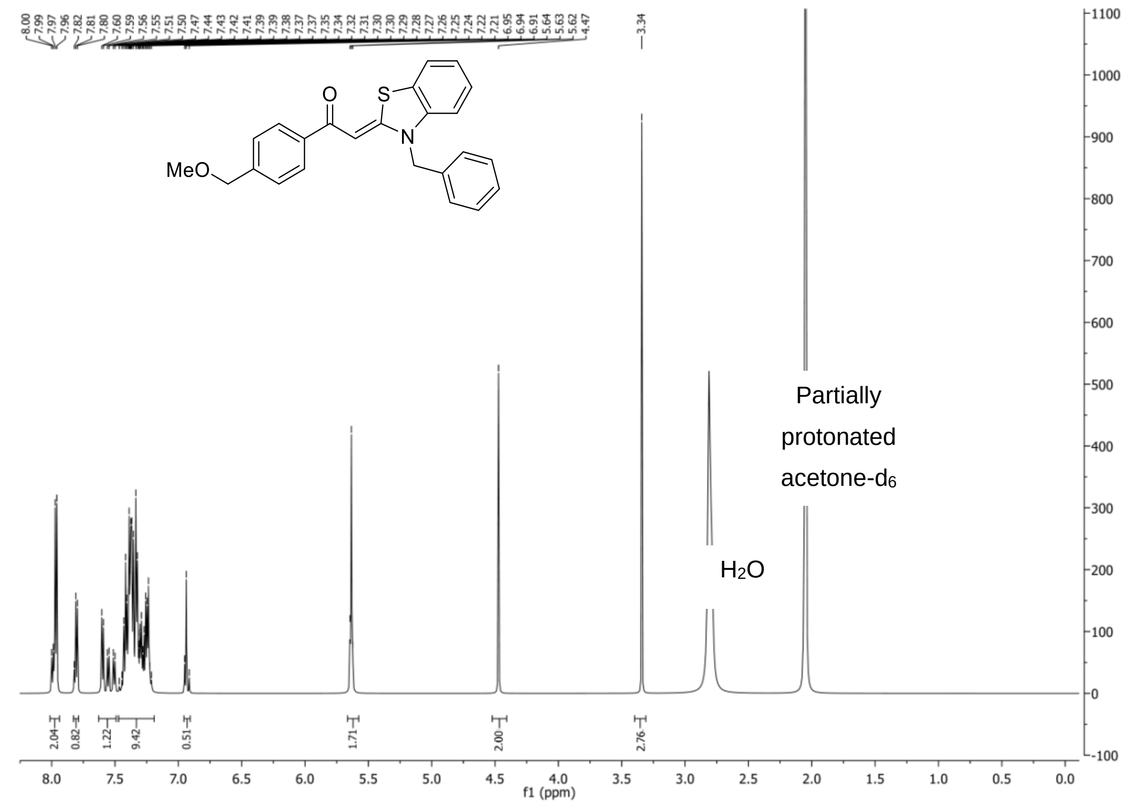

Figure S15. ^{1}H NMR spectrum (*Z*)-2-(3-benzylbenzo[*d*]thiazol-2(3*H*)-ylidene)-1-(4-(methoxymethyl)phenyl)ethan-1-one (**4h**) (600 MHz, acetone-d$_6$, 293 K).

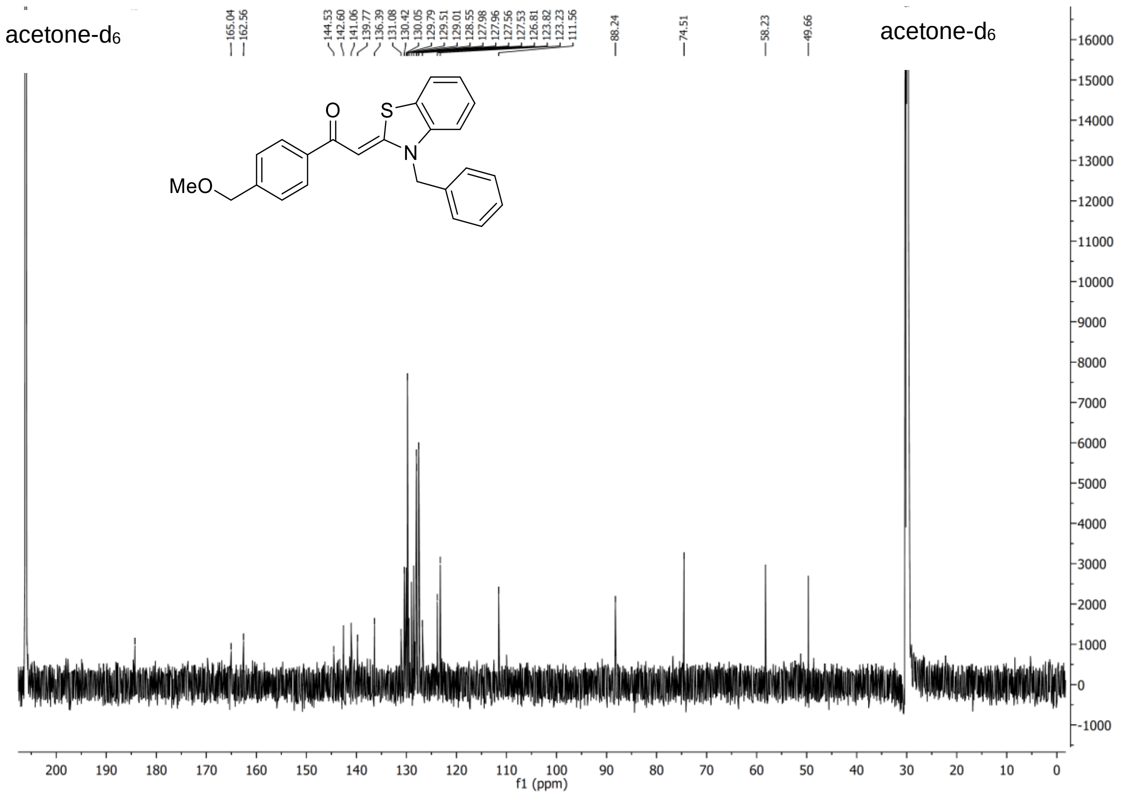

Figure S16. ^{13}C NMR spectrum (*Z*)-2-(3-benzylbenzo[*d*]thiazol-2(3*H*)-ylidene)-1-(4-(methoxymethyl)phenyl)ethan-1-one (**4h**) (150 MHz, acetone-d$_6$, 293 K).

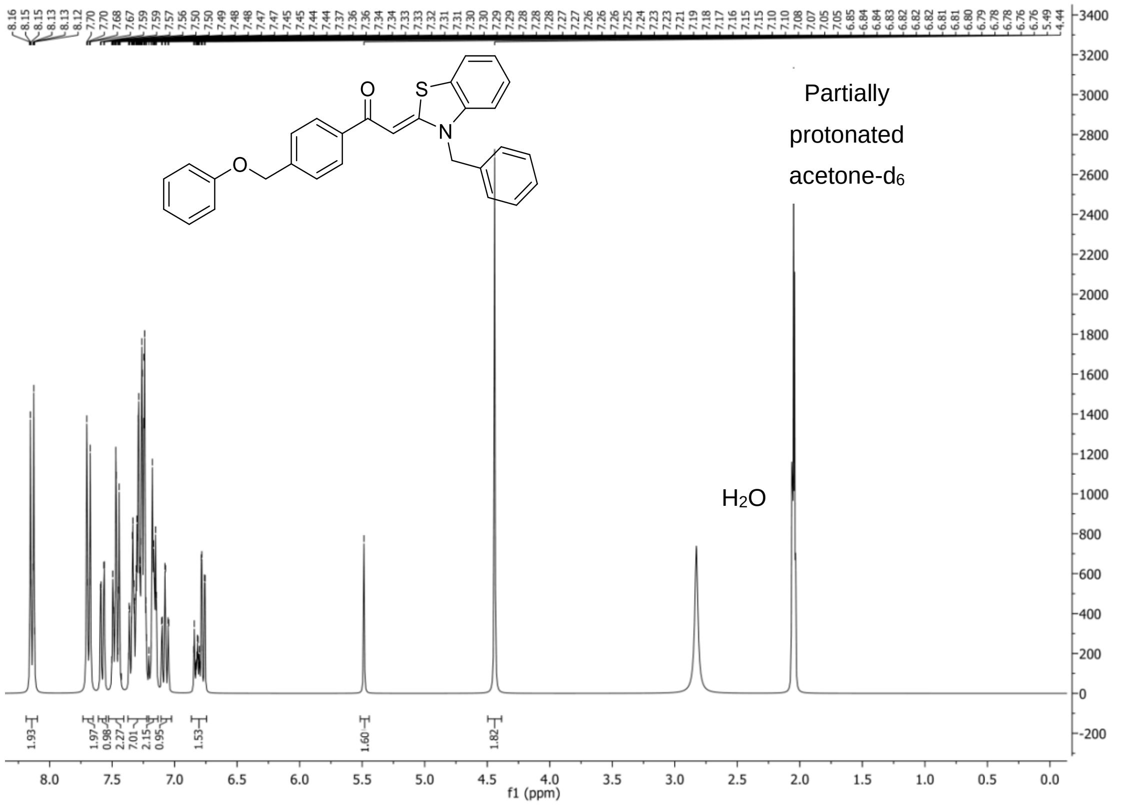

Figure S17. ^{1}H NMR spectrum (*Z*)-2-(3-benzylbenzo[*d*]thiazol-2(3*H*)-ylidene)-1-(4-(phenoxymethyl)phenyl)ethan-1-one (**4i**) (300 MHz, acetone-d_6, 293 K).

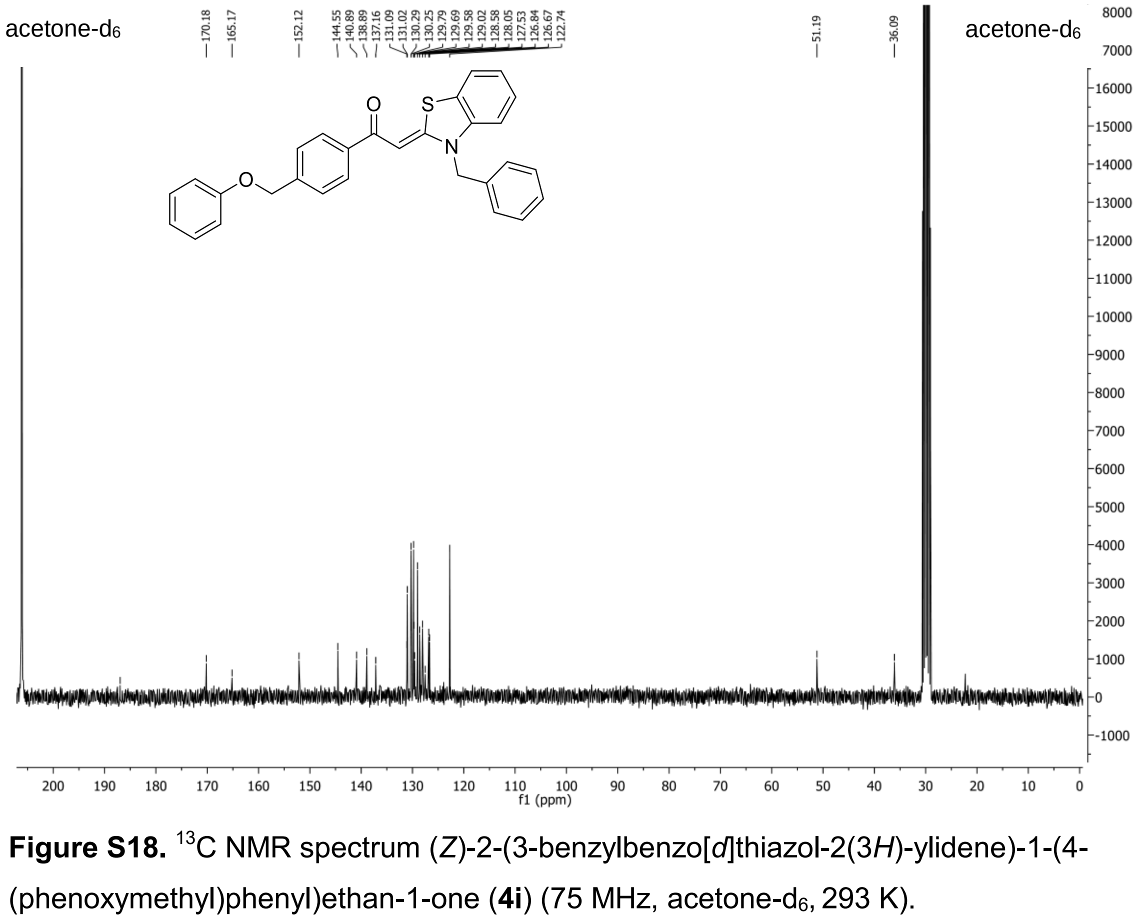

Figure S18. ^{13}C NMR spectrum (*Z*)-2-(3-benzylbenzo[*d*]thiazol-2(3*H*)-ylidene)-1-(4-(phenoxymethyl)phenyl)ethan-1-one (**4i**) (75 MHz, acetone-d_6, 293 K).

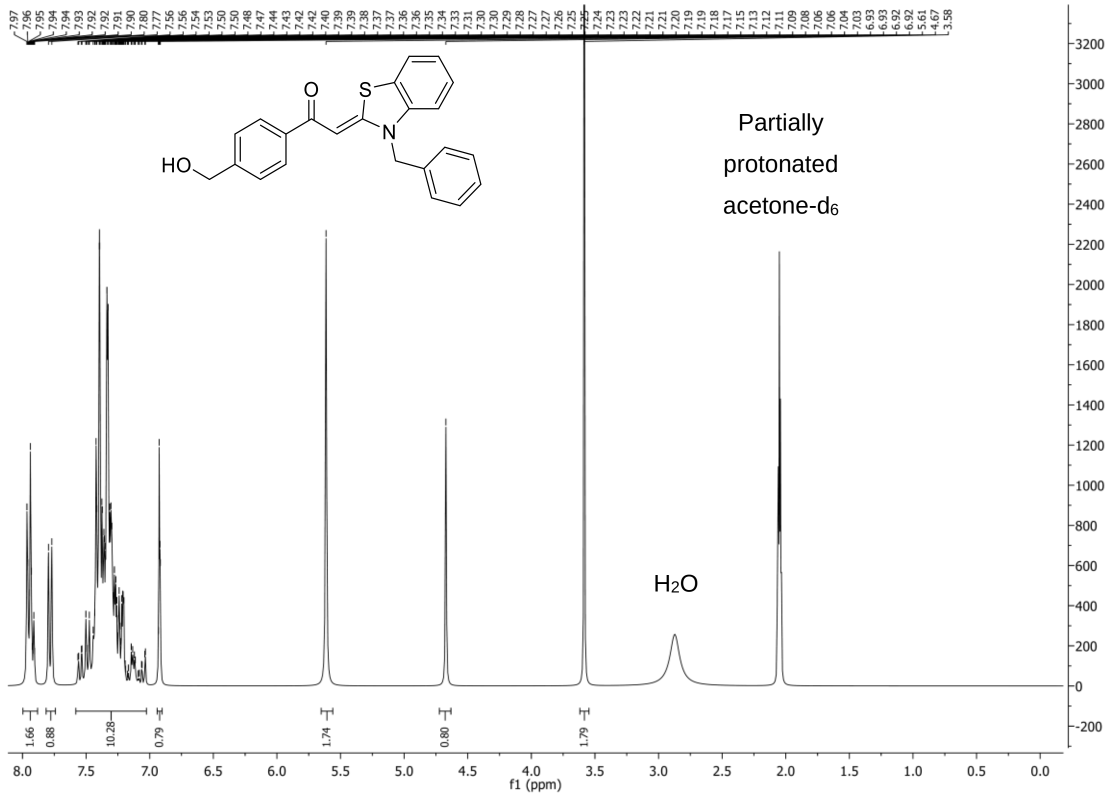

Figure S19. ^{1}H NMR spectrum (*Z*)-2-(3-benzylbenzo[*d*]thiazol-2(3*H*)-ylidene)-1-(4-(hydroxymethyl)phenyl)ethan-1-one (**4j**) (300 MHz, acetone-d_6, 293 K).

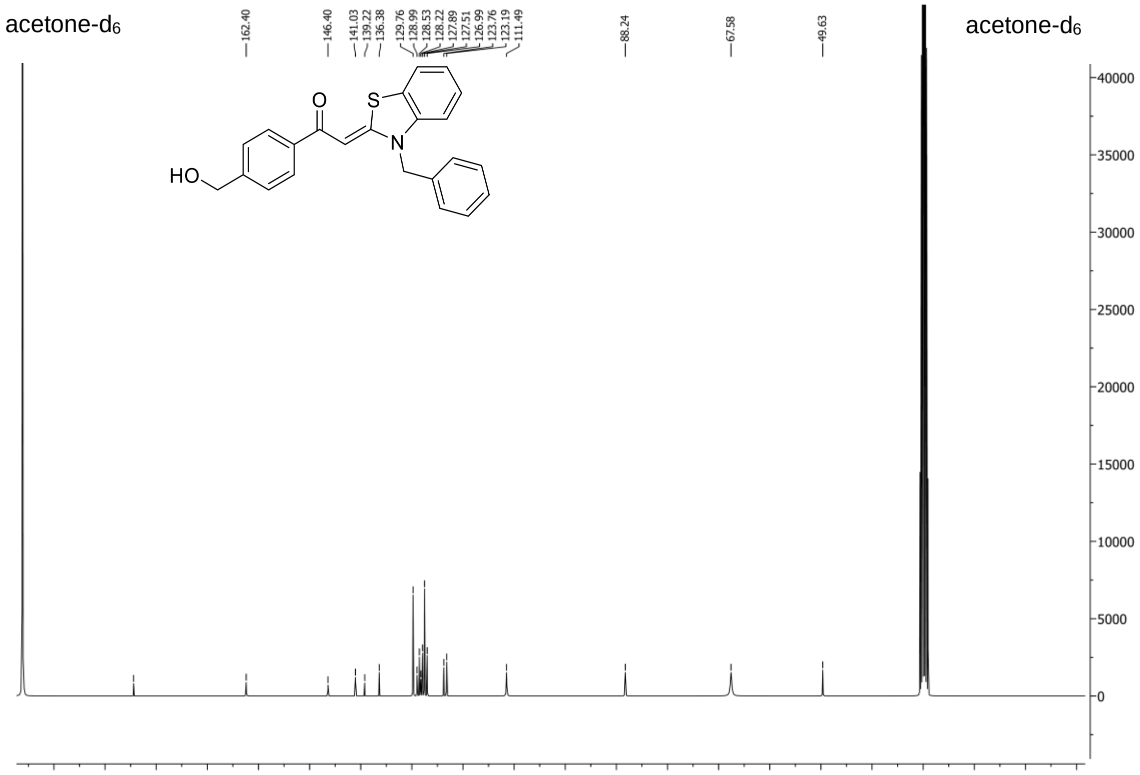

Figure S20. ^{13}C NMR spectrum (*Z*)-2-(3-benzylbenzo[*d*]thiazol-2(3*H*)-ylidene)-1-(4-(hydroxymethyl)phenyl)ethan-1-one (**4j**) (75 MHz, acetone-d_6, 293 K).

4 Overview of photophysical properties of etherified aroyl-*S,N*-ketene acetals 4

All solution spectra were recorded in ethanol or in ethanol/water mixtures at T = 298 K, the excitation wavelengths λ_{exc} for the AIE-titration studies and the emission spectra in solution were determined from the absorption maxima λ_{max} of this compound, the excitation wavelength for the solid-state emission spectra was determined from solid-state excitation spectra. The dye concentration of the solution for absorption measurements was c (**4**) = 10^{-5} M and the dye concentration of the ethanol/water mixtures for AIE measurements was c (**4**) = 10^{-7} M.

Table S3: Photophysical properties of etherified aroyl-*S,N*-ketene acetals **4**.

Entry	Compound	$\lambda_{max(abs.)}$ solution [nm] (ε [L·mol^{-1}·cm^{-1}])[a]	$\lambda_{max(em.)}$ solid-state [nm][b]	Quantum yield Φ_f	solid-state picture[c]
1	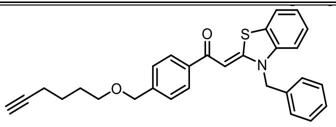 **4a**	385 (6700)	-	-	-
2	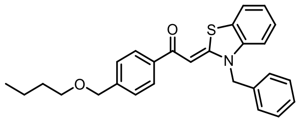 **4b**	386 (31100)	-	-	-
3	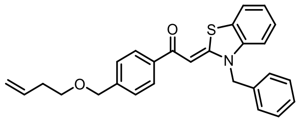 **4c**	387 (35300)	547	0.02	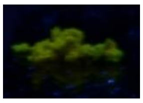
4	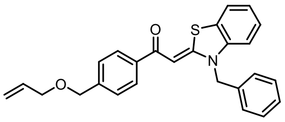 **4d**	386 (30700)	598	0.03	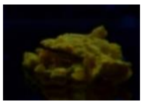
5	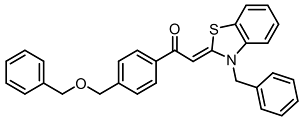 **4e**	385 (1600)	-	-	-

[a]: measured in ethanol, T = 298 K, c (**4**) = 10^{-5} M, [b]: T = 298 K, $\lambda_{exc} = \lambda_{abs,max}$, [c]: pictures taken under UV-light (λ_{exc} = 365 nm).

Table S3 continued.

Entry	Compound	$\lambda_{max(abs.)}$ solution [nm] (ε [L·mol^{-1}·cm^{-1}])[a]	$\lambda_{max(em.)}$ solid-state [nm][b]	Quantum yield Φ_f	solid-state picture[c]
6	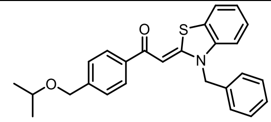 **4f**	386 (41300)	500	0.07	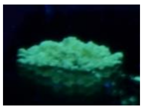
7	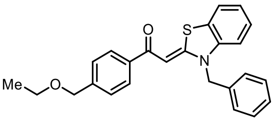 **4g**	385 (39000)	518	0.04	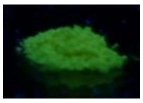
8	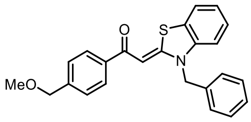 **4h**	385 (15500)	-	-	-
9	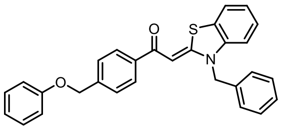 **4i**	385 (4600)	-	-	-
10	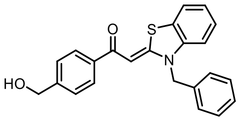 **4j**	256 (15300)<br>385 (22400)	618	0.04	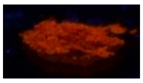

[a]: measured in ethanol, T = 298 K, c (**4**) = 10^{-5} M, [b]: T = 298 K, $\lambda_{exc} = \lambda_{abs,max}$, [c]: pictures taken under UV-light (λ_{exc} = 365 nm).

5 Absorption and emission spectra

All solution spectra were recorded in ethanol or in ethanol/water mixtures at T = 298 K, the excitation wavelengths λ_{exc} for the AIE-titration studies and the emission spectra in solution were determined from the absorption maxima λ_{max} of this compound, the excitation wavelength for the solid-state emission spectra was determined from solid-state excitation spectra. The dye concentration of the solution for absorption measurements was c(**4**) = 10^{-5} M and the dye concentration of the ethanol/water mixtures for AIE measurements was c(**4**) = 10^{-7} M.

5.1 Absorption and emission spectra of etherified aroyl-*S,N*-ketene acetals 4

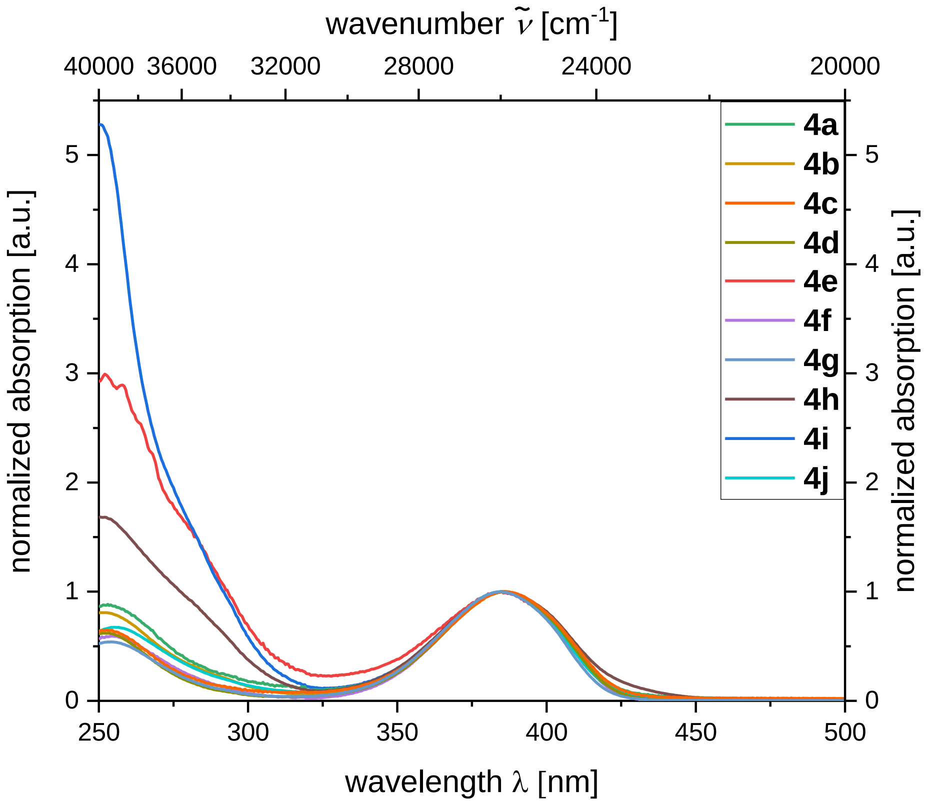

Figure S21: Selected, normalized UV/Vis absorption and emission bands of etherified aroyl-*S,N*-ketene acetals **4.**

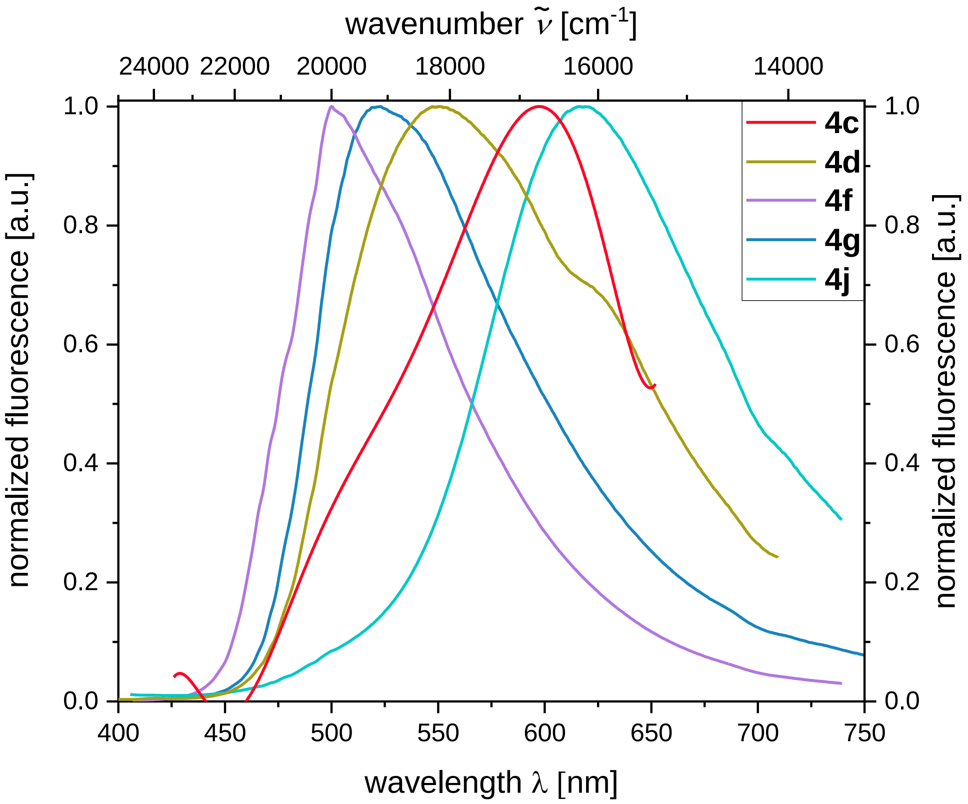

Figure S22: Selected, normalized solid-state emission bands of etherified aroyl-*S,N*-ketene acetals **4**.

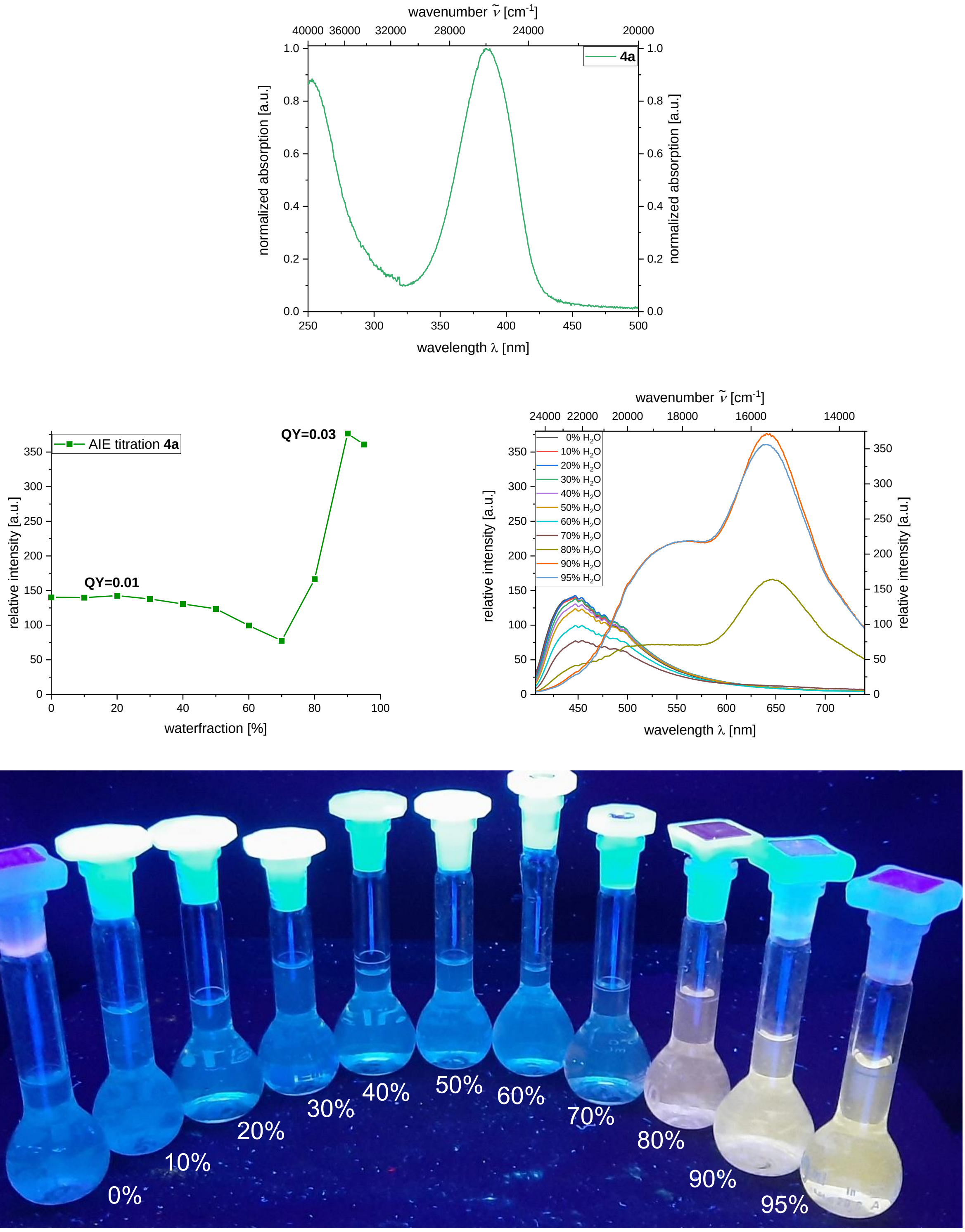

Figure S23: Absorption spectrum of **4a** in ethanol (top), and AIE-induced changes in emission (center, left), AIE-related emission spectra of compound **4a** (center, right) and photographs of solutions of dye **4a** in ethanol/water mixtures of increasing water content (bottom). The latter spectra were measured in ethanol/water mixtures of varying water content.

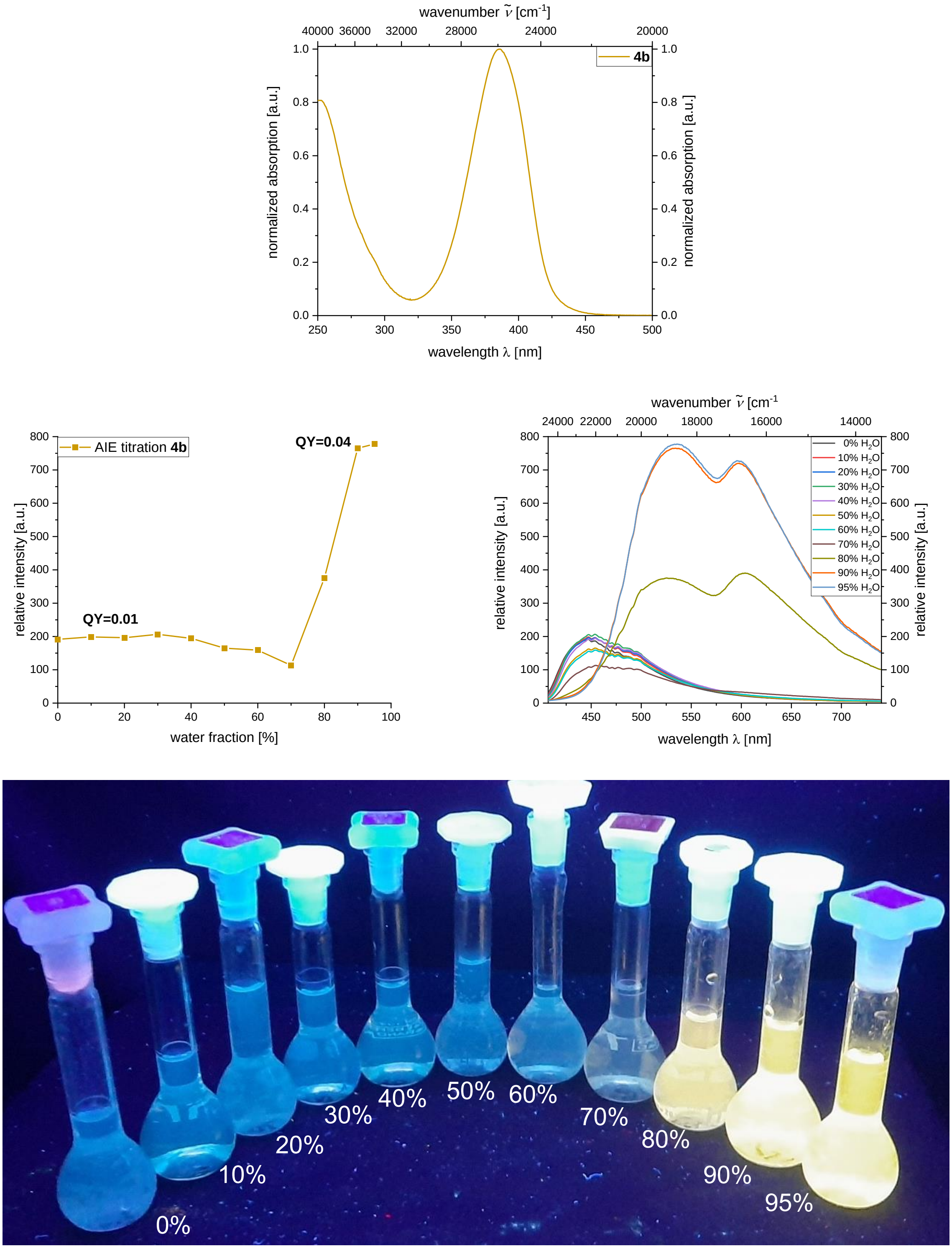

Figure S24: Absorption spectrum of **4b** in ethanol (top), , and AIE-induced changes in emission (center, left), AIE-related emission spectra of compound **4b** (center, right) and photographs of solutions of dye **4b** in ethanol/water mixtures of increasing water content (bottom). The latter spectra were measured in ethanol/water mixtures of varying water content.

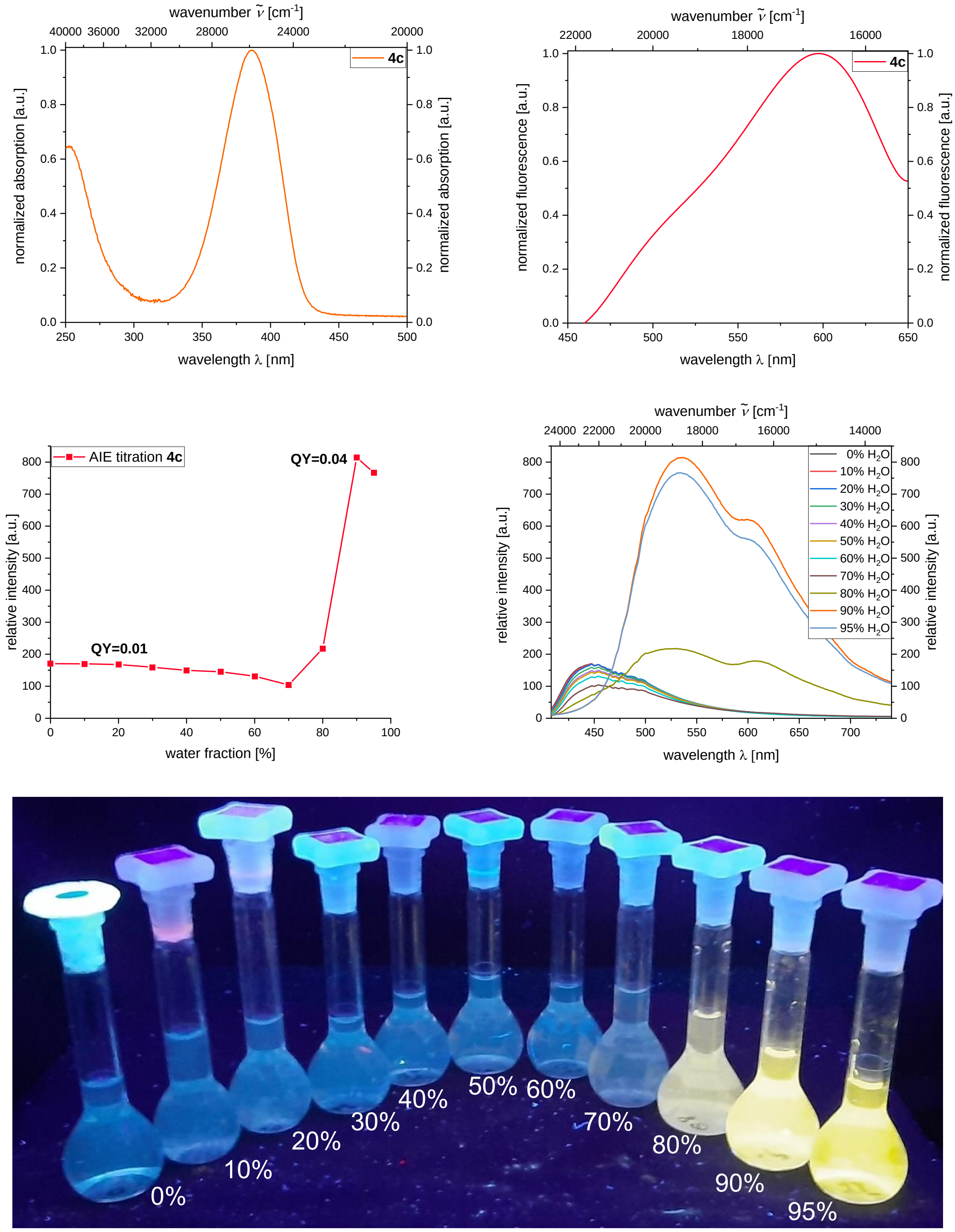

Figure S25: Absorption spectrum of **4c** in ethanol (top, left), solid-state emission spectrum (top, right), and AIE-induced changes in emission (center, left), AIE-related emission spectra of compound **4c** (center, right) and photographs of solutions of dye **4c** in ethanol/water mixtures of increasing water content (bottom). The latter spectra were measured in ethanol/water mixtures of varying water content.

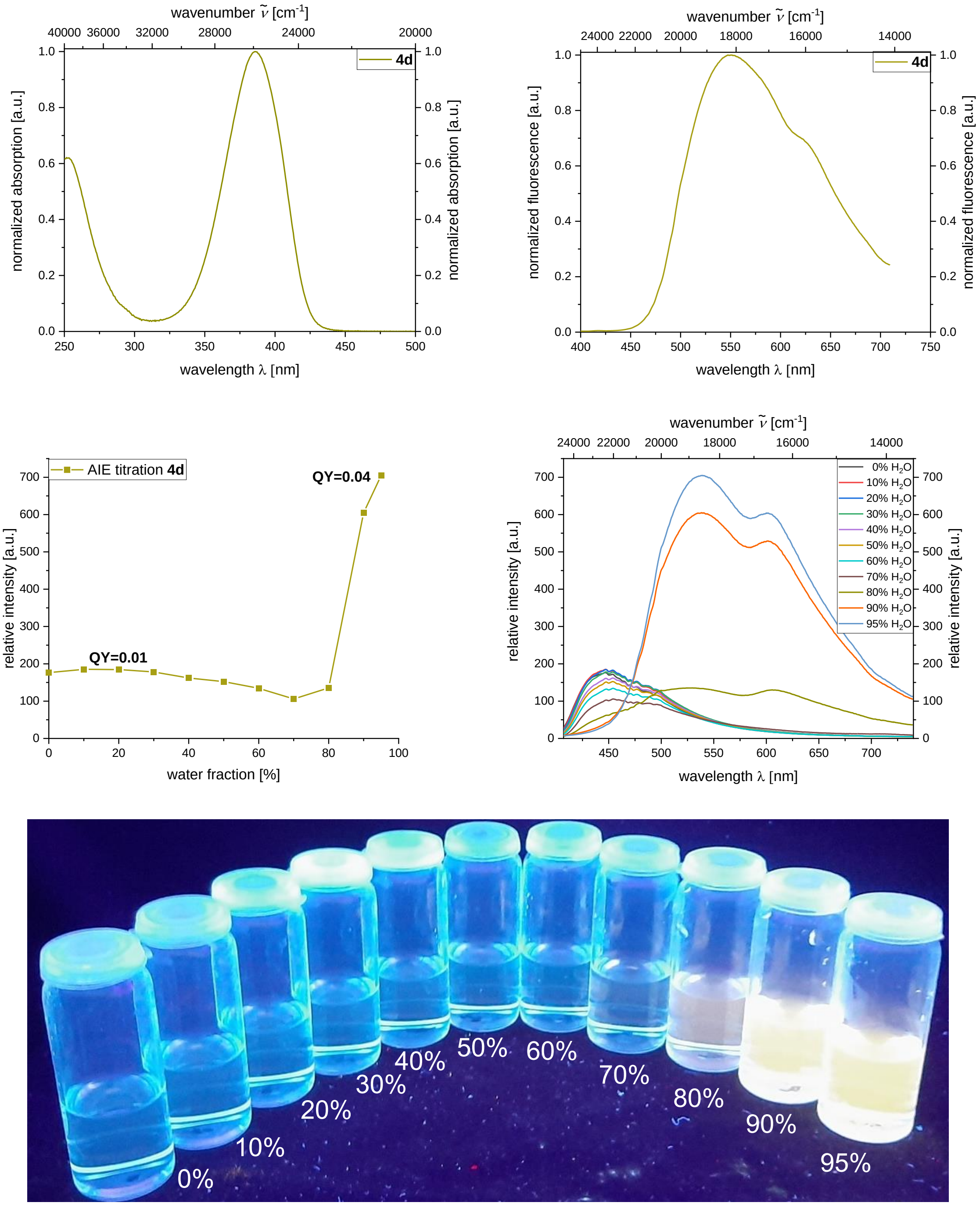

Figure S26: Absorption spectrum of **4d** in ethanol (top, left), solid-state emission spectrum (top, right), and AIE-induced changes in emission (center, left), AIE-related emission spectra of compound **4d** (center, right) and photographs of solutions of dye **4d** in ethanol/water mixtures of increasing water content (bottom). The latter spectra were measured in ethanol/water mixtures of varying water content.

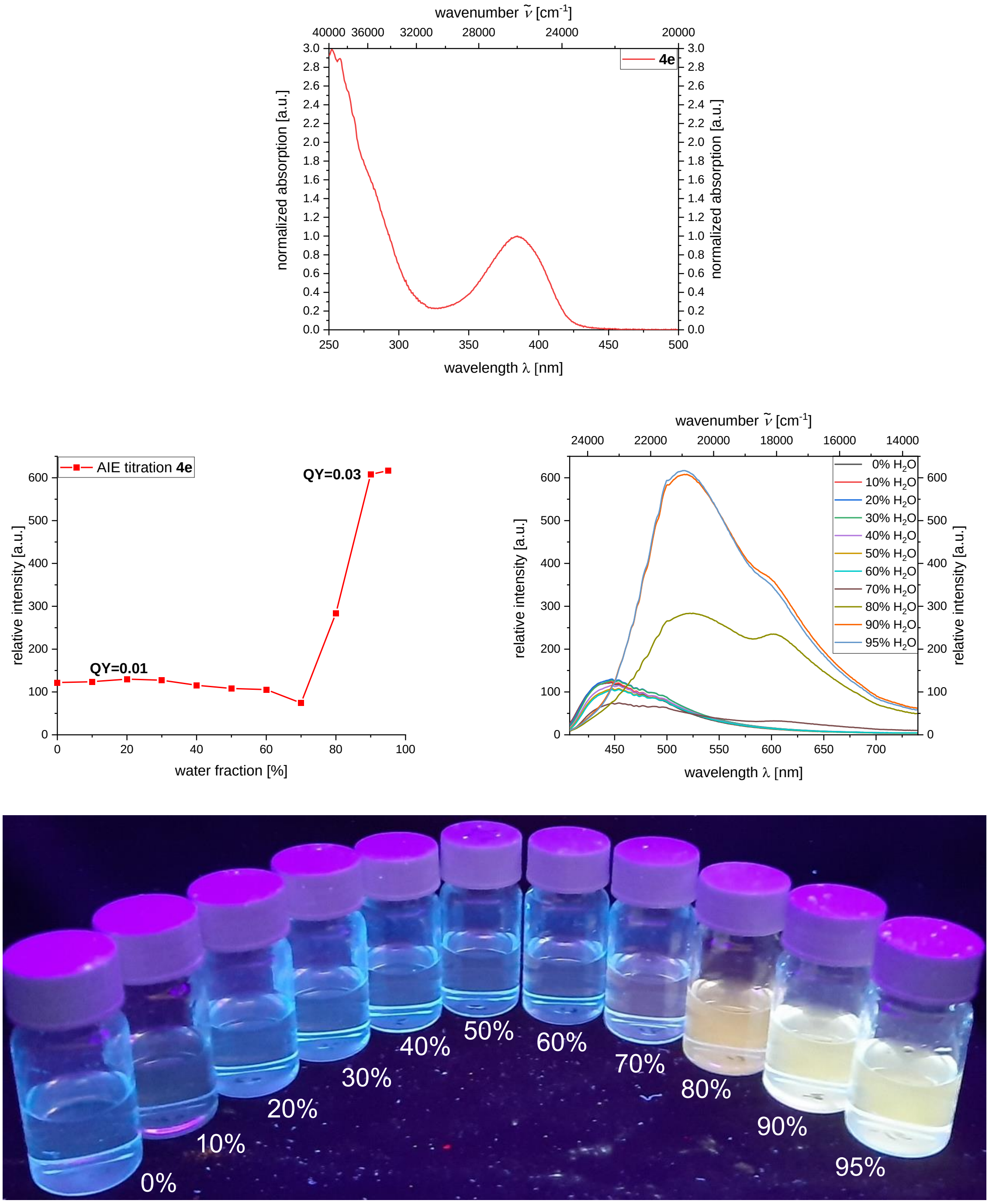

Figure S27: Absorption spectrum of **4e** in ethanol (top), and AIE-induced changes in emission (center, left), AIE-related emission spectra of compound **4e** (center, right) and photographs of solutions of dye **4e** in ethanol/water mixtures of increasing water content (bottom). The latter spectra were measured in ethanol/water mixtures of varying water content.

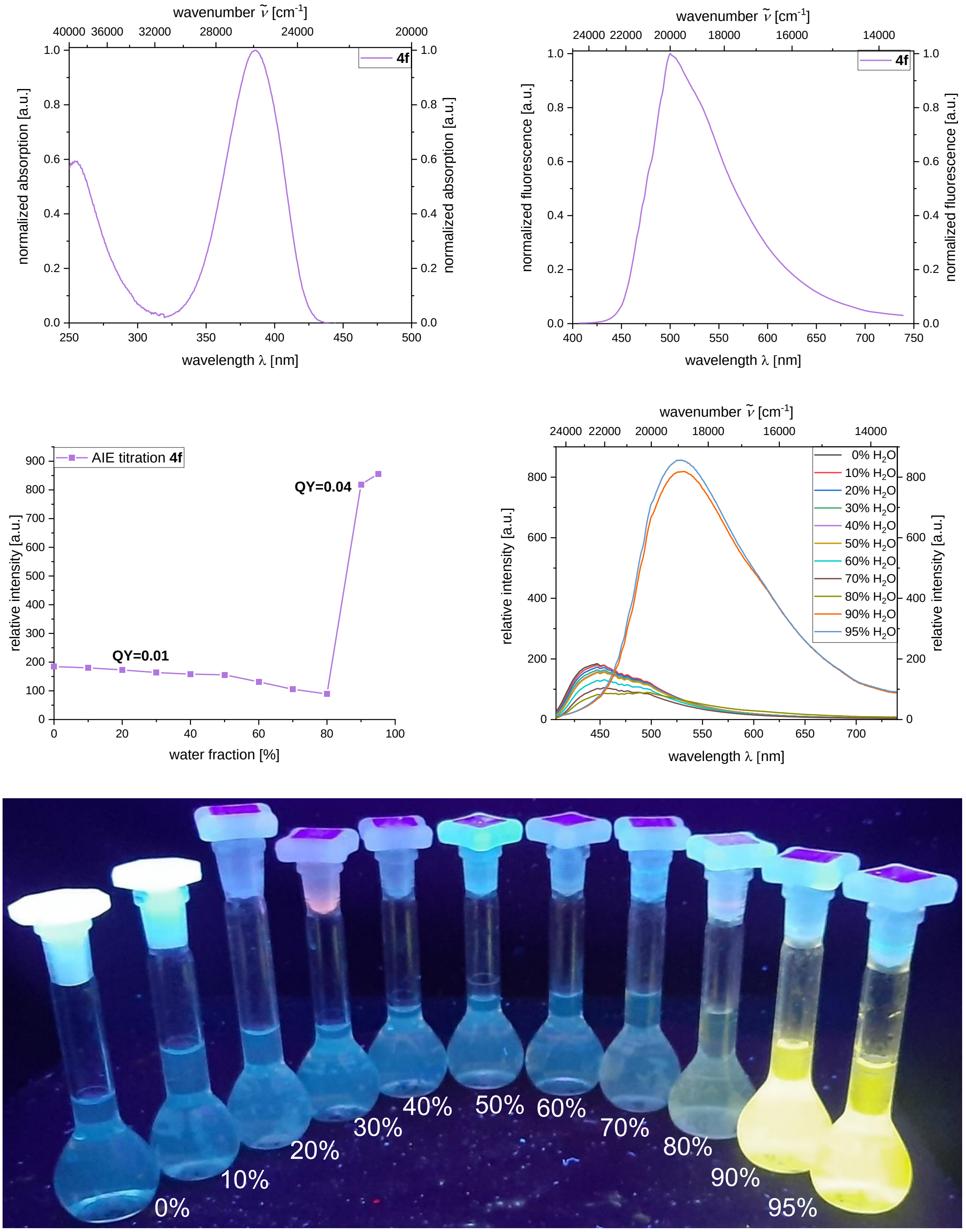

Figure S28: Absorption spectrum of **4f** in ethanol (top, left), solid-state emission spectrum (top, right), and AIE-induced changes in emission (center, left), AIE-related emission spectra of compound **4f** (center, right) and photographs of solutions of dye **4f** in ethanol/water mixtures of increasing water content (bottom). The latter spectra were measured in ethanol/water mixtures of varying water content.

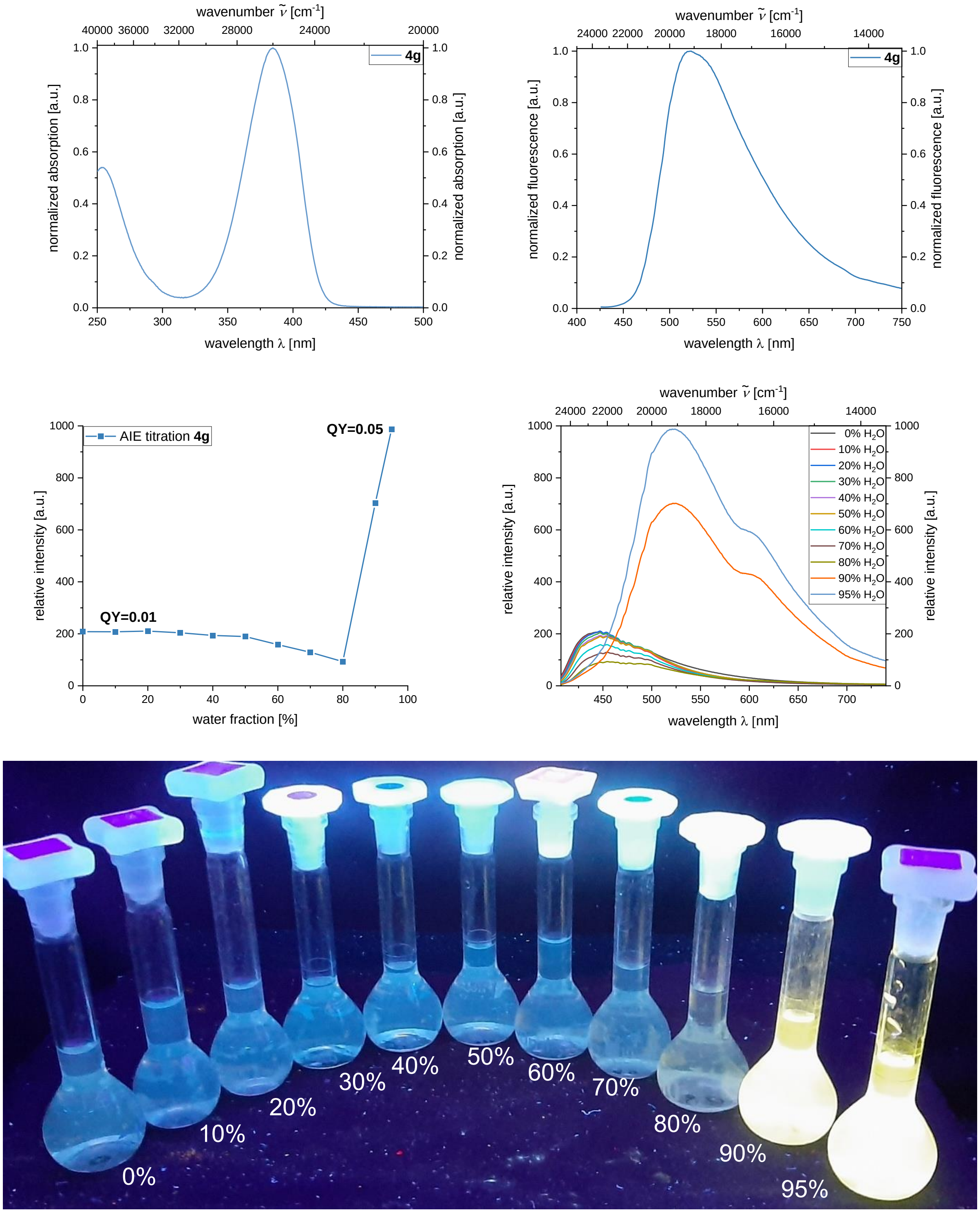

Figure S29: Absorption spectrum of **4g** in ethanol (top, left), solid-state emission spectrum (top, right), and AIE-induced changes in emission (center, left), AIE-related emission spectra of compound **4g** (center, right) and photographs of solutions of dye **4g** in ethanol/water mixtures of increasing water content (bottom). The latter spectra were measured in ethanol/water mixtures of varying water content.

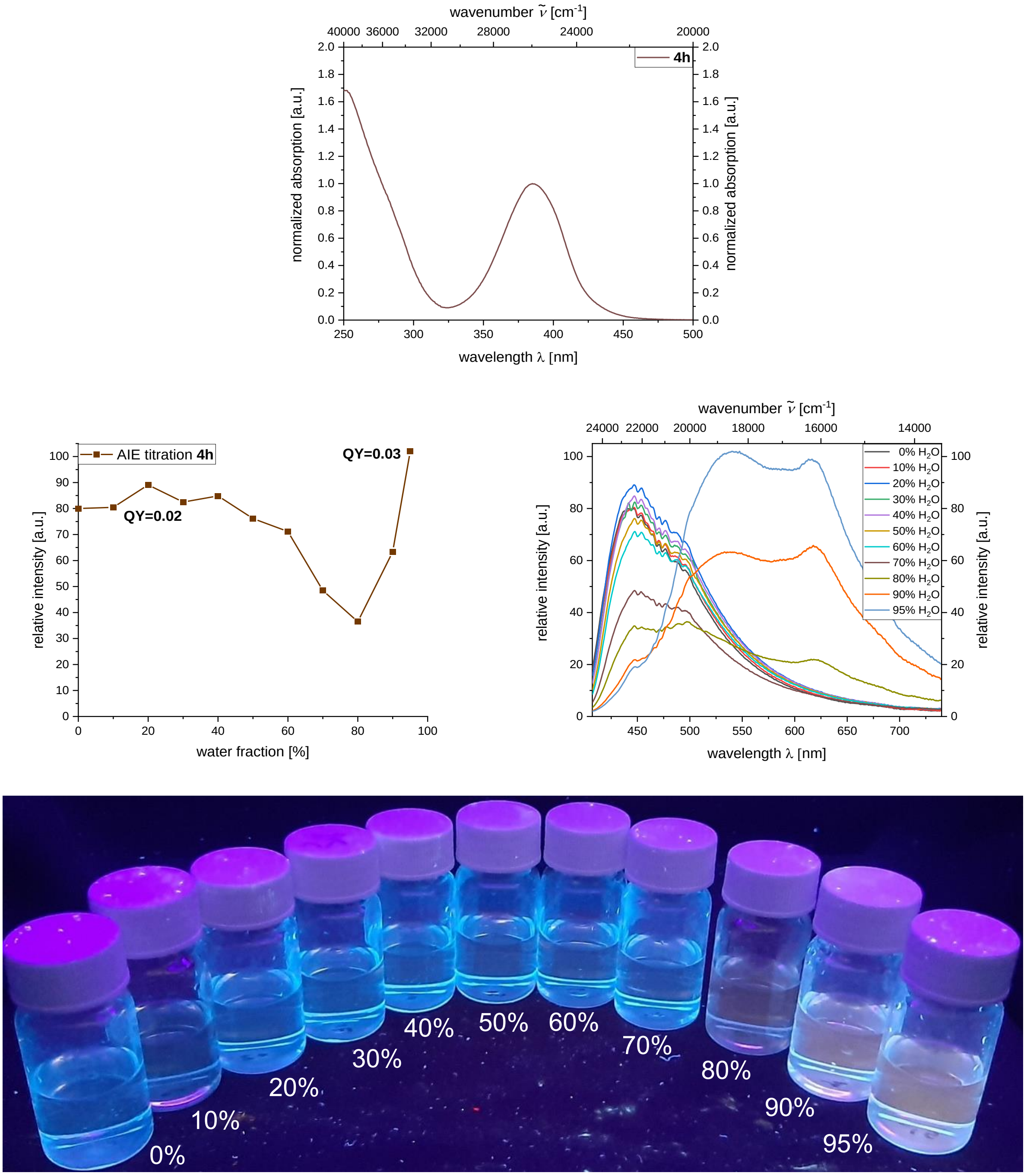

Figure S30: Absorption spectrum of **4h** in ethanol (top), and AIE-induced changes in emission (center, left), AIE-related emission spectra of compound **4h** (center, right) and photographs of solutions of dye **4h** in ethanol/water mixtures of increasing water content (bottom). The latter spectra were measured in ethanol/water mixtures of varying water content.

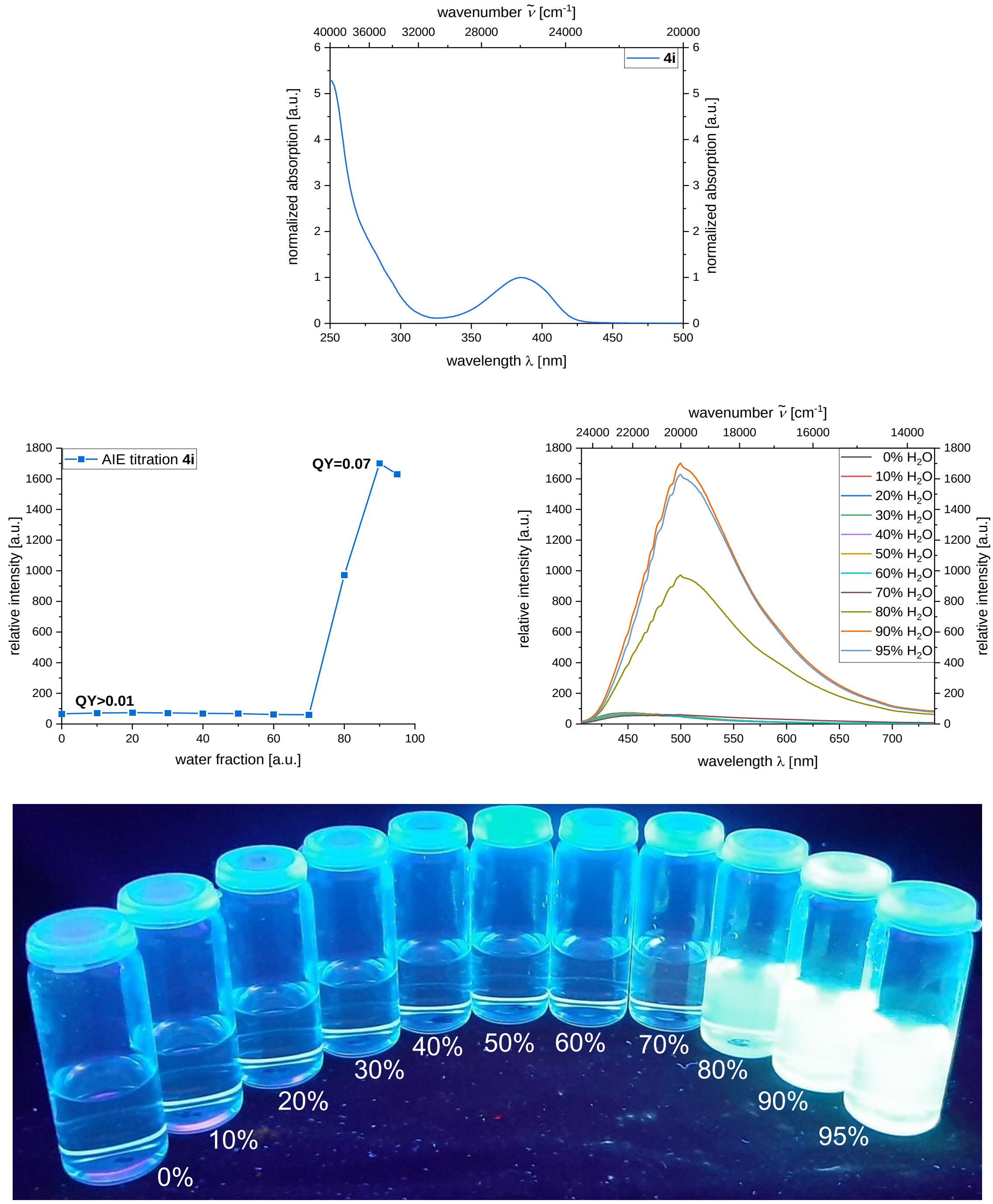

Figure S31: Absorption spectrum of **4i** in ethanol (top), and AIE-induced changes in emission (center, left), AIE-related emission spectra of compound **4i** (center, right) and photographs of solutions of dye **4i** in ethanol/water mixtures of increasing water content (bottom). The latter spectra were measured in ethanol/water mixtures of varying water content.

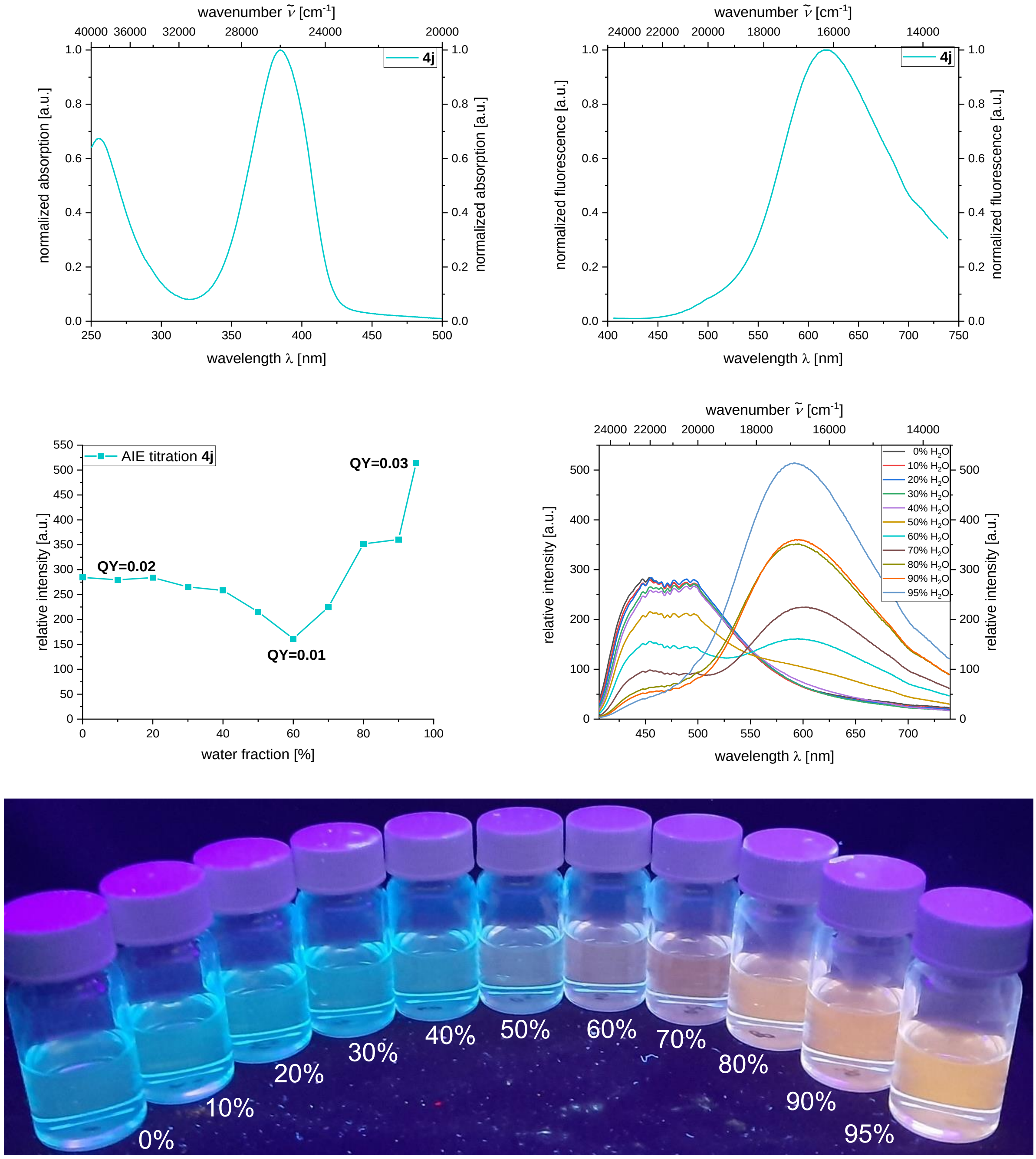

Figure S32: Absorption spectrum of **4j** in ethanol (top, left), solid-state emission spectrum (top, right), and AIE-induced changes in emission (center, left), AIE-related emission spectra of compound **4j** (center, right) and photographs of solutions of dye **4j** in ethanol/water mixtures of increasing water content (bottom). The latter spectra were measured in ethanol/water mixtures of varying water content.

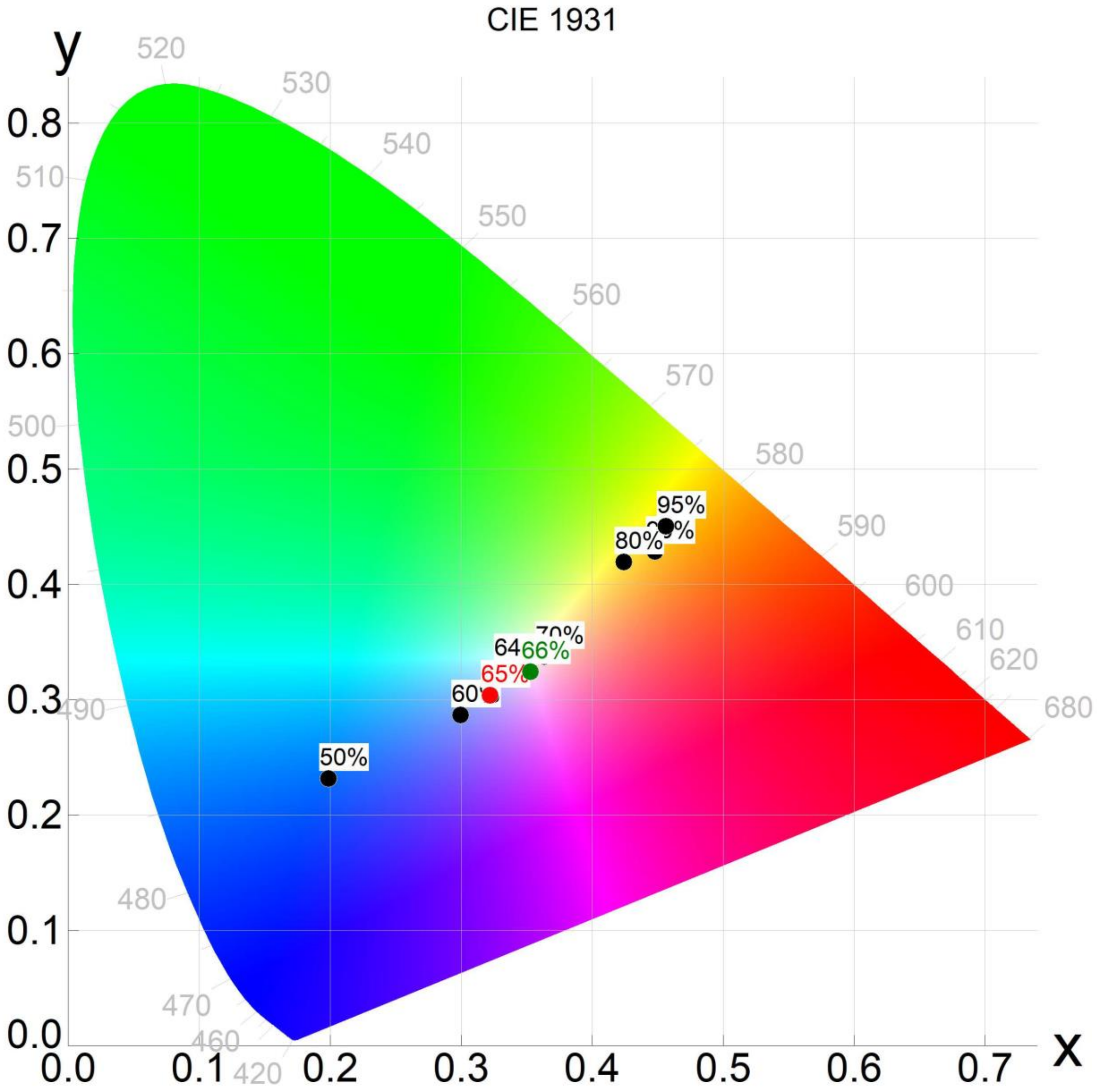

Figure S33: CIE scheme for selected water fractions AIE-related emission spectra of compound **4j.**

6 Literature

[1]: M. Kuhnert-Brandstatter, *Sci. Pharm.* **1966**, *34*, 147-166.

[2]: M. Hesse, H. Meier, B. Zeeh, *Spektroskopische Methoden in der organischen Chemie*, Georg Thieme Verlag, Stuttgart, **2005**.

[3]: S. K. Yen, L. L. Koh, F. E. Hahn, H. V. Huynh, T. A. Hor, *Organomet.* **2006**, *25*, 5105-5112.

[4]: L. Biesen, N. Nirmalananthan-Budau, K. Hoffmann, U. Resch-Genger, T. J. J. Müller, *Angew. Chem. Int. Ed*. **2020**, *59*, 1-6.

[5]: L. Biesen, D. Woschko, C. Janiak, T. J. J. Müller, *Chem. Eur. J.* **2022**, *28*, e202202579.
